# Replication Stress Is an Actionable Genetic Vulnerability in Desmoplastic Small Round Cell Tumors

**DOI:** 10.1158/0008-5472.CAN-23-3603

**Published:** 2024-10-16

**Authors:** Asuka Kawai-Kawachi, Madison M. Lenormand, Clémence Astier, Noé Herbel, Meritxell B. Cutrona, Carine Ngo, Marlène Garrido, Thomas Eychenne, Nicolas Dorvault, Laetitia Bordelet, Feifei Song, Ryme Bouyakoub, Anastasia Loktev, Antonio Romo-Morales, Clémence Henon, Léo Colmet-Daage, Julien Vibert, Marjorie Drac, Rachel Brough, Etienne Schwob, Oliviano Martella, Guillaume Pinna, Janet M. Shipley, Sibylle Mittnacht, Astrid Zimmermann, Aditi Gulati, Olivier Mir, Axel Le Cesne, Matthieu Faron, Charles Honoré, Christopher J. Lord, Roman M. Chabanon, Sophie Postel-Vinay

**Affiliations:** 1 The ATIP-Avenir Inserm and ERC StG (Epi)genetic Vulnerabilities in Solid Tumors and Sarcoma Laboratory, Inserm Unit UMR 981, Université Paris-Saclay, Gustave Roussy, Villejuif, France.; 2 Cancer RNA Research Unit, National Cancer Center Research Institute, Tokyo, Japan.; 3 Department of Genomes and Genetics, Institut Pasteur, CNRS UMR3525, Paris, France.; 4 Université Paris-Saclay, Université Paris-Sud XI, Faculté de Médicine, Le Kremlin Bicêtre, France.; 5 Drug Development Department, DITEP, Gustave Roussy, Villejuif, France.; 6 Sarcoma Committee, Gustave Roussy, Villejuif, France.; 7 Viroxis SAS Biotech, Gustave Roussy, Villejuif, France.; 8 Organoid Core Facility, Gustave Roussy, Villejuif, France.; 9 Experimental and Translational Pathology (PETRA) Platform, AMMICa Unit (CNRS Unit UMS 3655, Inserm Unit US 23), Gustave Roussy, Villejuif, France.; 10 The CRUK Gene Function Laboratory and Breast Cancer Now Toby Robins Research Centre, The Institute of Cancer Research, London, United Kingdom.; 11 Sarcoma Molecular Pathology Team, Divisions of Molecular Pathology and Cancer Therapeutics, The Institute of Cancer Research, London, United Kingdom.; 12 Institute of Molecular Genetics, CNRS Unit UMR 5535, Université de Montpellier, Montpellier, France.; 13 RNA Interference Platform PARi, IRCM/IBFJ/CEA UMRE008, Fontenay-aux-Roses, France.; 14 UCL Cancer Institute, University College London, London, United Kingdom.; 15 Research Unit Oncology, The Healthcare Business of Merck KGaA, Darmstadt, Germany.

## Abstract

Desmoplastic small round cell tumor (DSRCT) is an aggressive sarcoma subtype that is driven by the EWS–WT1 chimeric transcription factor. The prognosis for DSRCT is poor, and major advances in treating DSRCT have not occurred for over two decades. To identify effective therapeutic approaches to target DSRCT, we conducted a high-throughput drug sensitivity screen in a DSRCT cell line assessing chemosensitivity profiles for 79 small-molecule inhibitors. DSRCT cells were sensitive to PARP inhibitors (PARPi) and ataxia–telangiectasia and Rad3–related inhibitors (ATRi), as monotherapies and in combination. These effects were recapitulated using multiple clinical PARPi and ATRi in three biologically distinct, clinically relevant models of DSRCT, including cell lines, a patient-derived xenograft–derived organoid model, and a cell line–derived xenograft mouse model. Mechanistically, exposure to a combination of PARPi and ATRi caused increased DNA damage, G_2_–M checkpoint activation, micronuclei accumulation, replication stress, and R-loop formation. EWS–WT1 silencing abrogated these phenotypes and was epistatic with exogenous expression of the R-loop resolution enzyme RNase H1 in reversing sensitivity to PARPi and ATRi monotherapies. The combination of PARPi and ATRi also induced EWS–WT1–dependent cell-autonomous activation of the cyclic GMP–AMP synthase–stimulator of IFN genes innate immune pathway and cell-surface expression of PD-L1. Taken together, these findings point toward a role for EWS–WT1 in generating R-loop–dependent replication stress that leads to a targetable vulnerability, providing a rationale for the clinical assessment of PARPi and ATRi in DSRCT.

**Significance:**

EWS–WT1, the unique oncogenic driver of desmoplastic small round cell tumors, confers sensitivity to PARP and ATR inhibitors, supporting the potential of these drugs in treating patients with this aggressive sarcoma subtype.

## Introduction

Desmoplastic small round cell tumor (DSRCT) is a rare and aggressive subtype of sarcoma, affecting predominantly young males (1). DSRCT classically presents as a large abdominal mass and is most often diagnosed at advanced or metastatic stages with multiple peritoneal metastatic nodules and sometimes distant metastases. Clinical prognosis for advanced DSRCT remains poor with a 5-year survival rate below 15% (2). No major therapeutic advance has occurred for DSRCT over the past 20 years, and currently, patients with DSRCT undergo a standard Ewing sarcoma regimen, consisting of highly aggressive polychemotherapy and extensive surgical debulking (1). Therefore, the development of novel therapeutic strategies is urgently needed.

DSRCT is molecularly characterized by the t(11;22)(q13;q12) chromosomal translocation, which fuses the transactivation domain of *EWSR1* to the DNA-binding domain of *WT1*, encoding an aberrant chimeric transcription factor (2, 3). The presence of *EWSR1::WT1* rearrangement is pathognomonic of the disease and provides the diagnosis of DSRCT over other small round cell sarcomas (SRCS; ref. 2). Recent genomic sequencing identified rare additional secondary mutations, notably in genes encoding proteins involved in chromatin remodeling and DNA repair such as *ARID1A*, *KMT2C*, and *MSH3* (4–7). EWS–WT1 conditional expression in mesenchymal stem cells—the putative cell of origin of DSRCT—is necessary and sufficient to generate a DSRCT phenotype (8), and EWS–WT1 is considered the unique driver in this simple-genomics sarcoma (9). As such, this chimeric aberrant transcription factor represents the most evident therapeutic target in DSRCT. However, the direct targeting of transcription factors is extremely challenging (10), and one of the most promising strategies, which consists of degrading the target transcription factor, is just entering the clinic and has not yet been evaluated in transcription factor–driven sarcomas. Therefore, targeting downstream consequences of EWS–WT1 presence, such as transcription factor–induced oncogenic programs or replication stress, is an attractive strategy.

In this study, we aimed to identify novel actionable targeted dependencies in DSRCT, using functional genomics and small-molecule inhibitor screening. We found that two distinct DSRCT cell lines, one newly established patient-derived xenograft (PDX)–derived organoid (PDX-O) model and one cell line–derived xenograft mouse model, were selectively sensitive to PARP inhibitors (PARPi) and ataxia–telangiectasia and Rad3–related inhibitors (ATRi). Mechanistically, we found that the presence of EWS–WT1 increased DNA replication stress and R-loop formation, thereby causing enhanced reliance upon the ATR/CHK1 pathway. Exposure to PARPi and ATRi further activated the cyclic GMP–AMP synthase–stimulator of IFN genes (cGAS–STING) pathway and caused PD-L1 upregulation in DSRCT cell lines, suggesting potential for these drugs as DNA repair–targeted therapies and immunomodulators in DSRCT.

## Materials and Methods

### Cell lines

The DSRCT JN-DSRCT-1 (JN1) cell line was purchased from ATCC. The DSRCT R cell line was created in-house, derived from a PDX shared by Dr. Armelle Logié-Dishington (Champions Oncology, Hackensack, NJ). Briefly, the PDX was finely minced into tiny pieces that were subsequently washed in FBS and centrifuged. The pellet was resuspended in Dulbecco's Modified Eagle's Medium and Ham's F-12 nutrient mixture (DMEM/F-12) supplemented with 20% FBS, 1× Minimum Essential Medium (MEM) nonessential amino acids (#11140050, Gibco), and 1× penicillin/streptomycin (#15070063, Gibco) and incubated in a 10-cm^2^ Petri dish at 37°C and 5% CO_2_. The culture medium was changed every other day with recovery of suspended cells by centrifugation at 1,200 rpm. After 6 to 8 weeks, a partially homogeneous cell layer was obtained; from this primary culture, cells were washed with 1× PBS, dissociated in trypsin-EDTA solution (#25200056, Gibco), and seeded into a new culture flask for subsequent cell culture. JN1 and R cells were cultured in DMEM/F-12, supplemented with 10% or 20% of FBS, respectively. A673 and SaOS-2 cells were cultured in DMEM, supplemented with 10% FBS. All cells were grown at 37°C and 5% CO_2_. *Mycoplasma* testing was performed bimonthly using the MycoAlert Mycoplasma Detection Kit (Lonza). All cell lines were short tandem repeat typed using STEMELITE ID (Promega) to confirm identity.

The JN1 and R cell lines were originally derived from human tumors that were histopathologically diagnosed as DSRCT (11): the JN1 cell line was established from the pleural effusion of a 7-year-old male patient with metastatic DSRCT with the pathognomonic *EWSR1::WT1* fusion 3′-(CCC​ATG​GAT​GAA​GGA​CCA​GAT​CTT​GAT​CTA​G)-(GTG​AGA​AAC​CAT​ACC​AGT​GTG​ACT​TCA​AGG)-5′ (Supplementary Fig. S1); the R cell line was established from the lymph node of a 20-year-old male patient with metastatic DSRCT with the pathognomonic *EWSR1::WT1* fusion 3′-(GGA​GAG​CGA​GGT​GGC​TTC​AAT​AAG​CCT​GGT​G)-(GTG​AGA​AAC​CAT​ACC​AGT​GTG​ACT​TCA​AGG)-5′ (Supplementary Fig. S2). The Ewing sarcoma A673 cell line was gifted by Dr. Olivier Delattre (Institut Curie, Paris, France), and the osteosarcoma SaOS-2 cell line was gifted by Dr. Olivia Fromigue (Gustave Roussy, Villejuif, France).

### Generation of RNase H1–overexpressing JN1 cells

To generate stable RNase H1–expressing JN1, the ppyCAG-RNaseH1-V5 plasmid (Addgene, #111906) was transfected in JN1 cells with Lipofectamine 2000 (Thermo Fisher Scientific) according to the manufacturer’s instructions. Stable pools of transfectants were generated by selection with hygromycin B, and the resulting three selected populations were submitted to clonal isolation using the limiting dilution method. Clones were recovered and profiled for RNase H1 expression by Western blotting.

### Drugs and chemicals

PARPi olaparib (AZD2281), talazoparib (BMN-673), and veliparib (ABT-888); the ATRi gartisertib (M4344), ceralasertib (AZD6738), and berzosertib (M6620); and the CHK1 inhibitors (CHK1i) prexasertib (LY2606368) and SRA-737, as well as cisplatin, topotecan, and SN-38, were purchased from Selleck Chemicals. The ATRi tuvusertib (M1774) was provided by Merck. Inhibitor stock solutions were prepared in DMSO and stored in aliquots at −80°C. Mitomycin C, thymidine, iodo-deoxyuridine, and 5-chloro-2′-deoxyuridine were purchased from Sigma-Aldrich. PicoGreen was purchased from Thermo Fisher Scientific.

### Small-molecule inhibitor and drug screen

The small-molecule inhibitor and drug screen was performed as described previously (12). Briefly, small molecules were purchased as solid from suppliers listed in Supplementary Table S1 and stored in DMSO. Prior to the 384-well plate screen, solid small molecules were resuspended in DMSO as 10 mmol/L stocks, prior to further dilution in DMSO to create 384-well plates containing a titration (0.5, 1, 5, 10, 50, 100, 500, and 1,000 nmol/L). A Hamilton Microlab STAR liquid-handling platform was used for this and all subsequent liquid-handling steps, except for cell seeding.

JN1 cells growing in log phase were seeded in 384-well plates at 250 cells per well in 50 μL of culture medium using Thermo Fisher Scientific Multidrop Combi. This plating density was optimized to ensure that the cells were in growth phase by the end of the 5-day treatment. At 24 hours after seeding, the medium was removed and replaced with a medium containing the small-molecule inhibitor library, as detailed above. Cells were then continuously cultured in the presence of small-molecule inhibitors for a period of 5 days, at which point, cell viability was estimated by adding 20 μL of CellTiter-Glo (Promega), diluted 1:4 in PBS to the medium. After 10 minutes of incubation at room temperature, CellTiter-Glo–generated luminescence was captured using a VICTOR X light plate reader. Luminescence values from each well were normalized to the median of signals from wells exposed to DMSO only (in the absence of small-molecule inhibitors) to generate surviving fractions (SF). In total, the cell line was screened three times, generating triplicate SF datasets. SFs were then used to plot dose–response survival curves, which were generated using three-parameter logistic regression analysis via the drc package in R. Using drc, the AUC values were calculated from the dose–response survival curves. AUC values were expressed as the proportion of the maximum area, representing no response to a drug. They were further scaled to lie between 0 and 1. AUC values that were greater than 1 were capped at 1. Unscaled AUC values for each drug were also standardized, generating robust *Z*-scores based on the median AUC effect in a panel of 92 cancer cell lines (Supplementary Tables S2–S4) and the median absolute deviation of these effects. *Z*-scores were then plotted as a waterfall plot.

### Two-dimensional cell-based assays

Cells were plated in 96-well plates at 7,000 cells per well for JN1 cells and 10,000 cells per well for R cells and continuously exposed to drugs for a period of 7 days in culture. In the case of siRNA transfection, cells were transfected in 6-well plates 48 hours prior to drug exposure and trypsinized and reseeded at the density specified above in 96-well plates 24 hours prior to drug exposure. Cell viability was estimated by the addition of 50 μL of CellTiter-Glo Luminescent Cell Viability Assay (Promega), diluted in 1:4 in PBS. After 10-minute incubation at room temperature, the CellTiter-Glo–generated luminescence was captured using a VICTOR X light plate reader. Luminescence values from each well were normalized to the median signal of wells exposed to DMSO (vehicle) to generate SFs. SFs were then used to plot dose–response survival curves using GraphPad Prism.

For synergy analyses, cells were seeded in 96-well plates and continuously exposed to increasing concentrations of talazoparib (1:4 serial dilution, range: 0–500 nmol/L) and/or M4344 (1:3 serial dilution, range: 0–1,000 nmol/L) for 7 days in culture. Cell viability was assessed as described above. The median response of replicates was normalized per median marginal value (i.e., response in the absence of treatment). Synergy analysis was performed using R package SynergyFinder. Dose–response curves for single drugs were fitted to a four-parameter log-logistic model. Synergy scores were calculated using the Bliss independence model.

### Three-dimensional spheroid assay

To form spheroids, 500 JN1 cells in 200 μL of media were plated into each well of 96-well ultralow attachment plates (#7007, Corning). Once spheroids reached an area of ∼200,000 μm^2^, they were subjected to treatment with increasing concentrations of M6620 or SRA-737, in the presence or absence of SN-38 (at 0.25 or 0.5 nmol/L) for 5 days, with drug-containing medium replenishment after 3 days. At day 5, the media were removed and replaced with fresh media, and the spheroid size was monitored for up to 19 days from starting treatment, using a Celigo image cytometer (Revvity).

### Development of PDXs

The establishment of PDXs was conducted as previously described (13). All animal procedures and studies were performed in accordance with the approved guidelines for animal experimentation by the Ethics Committee at the Université Paris-Sud (CEEA 26, project 2014_055_2790) following EU regulation. Animals were housed under pathogen-free conditions with food and water *ad libitum*. At 1 to 12 hours after patient biopsy, fresh tumor fragments were implanted under the renal capsule of 6- to 8-week-old male NOD/SCID gamma (NSG) mice obtained from Charles River Laboratories.

### Derivation of DSRCT three-dimensional organoid cultures from a PDX tumor biopsy

A PDX model was first established from the primary peritoneal tumor of an 11-year-old male patient with DSRCT. From this PDX, a tumor biopsy was taken and divided into various pieces for downstream processing, including the derivation of DSRCT three-dimensional (3D) primary organoid cultures, referred to as GR_13 PDX-O. For cell dissociation, a sample of the biopsy (∼100 mm^3^) was preserved in tissue storage solution (#130-100-008, Miltenyi Biotec) at 4°C and processed in less than 1 hour. The sample was minced into small pieces that were subsequently digested in 5 mL of Hank’s Balanced Salt Solution buffer with calcium and magnesium (#24020091, Gibco), containing 7.4 mg/mL collagenase type II (#17101-015, Gibco) for 1 hour at 37°C. The digestion was stopped by adding 20 mL of Advanced DMEM/F-12 (#12634028, Gibco) supplemented with 1× penicillin/streptomycin (#15070063, Gibco) and 10% FBS (#SV30160.03, HyClone). The homogenate was passed through a 100-μm cell strainer (#542000, Greiner Bio-One) to remove debris and cell clumps, and the cell suspension was then centrifuged for 10 minutes at 450*g*. After aspiration of the supernatant, the cell pellet was resuspended in 1 mL of the abovementioned blocking medium.

To obtain human DSRCT cells and separate them from mouse cells, we used a cell depletion kit (#130-104-694, Miltenyi Biotec). Briefly, the cell suspension was centrifuged for 10 minutes at 450*g* and resuspended in 80 μL of PBS containing 0.5% w/v BSA. Mouse cells were magnetically labeled by incubating the cell suspension with 20 μL of mouse depletion cocktail for 15 minutes in the refrigerator. Human tumor cells were obtained from the flow-through, after passing the labeled cell suspension using magnetic separation and LS columns (#130-122-729, Miltenyi Biotec).

### PDX-O culture

The cells were counted and plated in 96-well U-bottom ultralow attachment wells (#7007, Corning; #650970, Greiner Bio-One) to ensure the formation of organoids in each well (5,000 viable cells in 100 μL of complete organoid medium per each well). The medium was refreshed every week by aspirating and adding 50 μL of complete organoid medium in each well, and the organoids were passaged every 3 to 4 weeks. The basal organoid medium formulation consisted of advanced DMEM/F-12 (#12634028) supplemented with 10 mmol/L HEPES (#15630049), 1% GlutaMAX (#35050038), 1× B27 supplement (#17504044), 1% penicillin/streptomycin (#15140122), and 1× N-2 Supplement (#17502048; all obtained from Thermo Fisher Scientific), 5% FBS (#F7524, Sigma-Aldrich), and 50 μg/mL Primocin (#Ant-pm-05) and 10 μg/mL Fungin (#Ant-fn-1; both obtained from InvivoGen). To obtain the complete organoid medium, the basal medium was supplemented with 1 mmol/L N-acetylcysteine (#A72250, Sigma-Aldrich), 10 mmol/L nicotinamide (#N0636, Sigma-Aldrich), 10 ng/mL recombinant human RSPO3 (#120-44, PeproTech), 10 ng/mL recombinant human Wnt3A (#HZ-1296, Proteintech), 10 ng/mL leukemia inhibitory factor (LIF; #HZ-1292, Proteintech), 25 ng/mL recombinant human IL22 (#HZ-1325, Proteintech), 10 pg/mL IL6 (#HZ-1019, Proteintech), 50 ng/mL recombinant human FGF-basic (#100-18B, PeproTech), and 100 ng/mL recombinant human insulin-like growth factor (IGF; #100-11, PeproTech). Then, 10 μmol/L ROCK Inhibitor Y-27632 (#S1049, Selleckchem) was added at the initial culture. The cells were plated using eight-channel VIAFLO electronic pipettes (#4624 and #4626, Integra). Finally, the plates were centrifuged for 5 minutes at 450*g*.

### PDX-O drug combination survival assay

After 3 weeks of culture, 240 GR_13 organoids were manually collected from the 96-well plates, transferred into an Eppendorf tube, and centrifuged for 5 minutes at 450*g*. The pelleted organoids were washed three times with 1× PBS and dissociated with TrypLE Express enzyme (#12604-013, Thermo Fisher Scientific). Next, the cells were filtered using a 70-μm cell strainer (#542070, Greiner Bio-One) and resuspended in complete organoid medium. For the drug combination survival assay, 4,000 cells were seeded in 40 μL of complete organoid medium per well in U-bottom 96-well plates (#4515, Corning). The formation of organoids was monitored for 3 days through bright-field acquisition every 24 hours using Incucyte SX1 (Sartorius), prior to adding the drugs. Serial fivefold dilutions of talazoparib or M4344 were prepared to yield final concentrations ranging from 50 μmol/L to 16 nmol/L (talazoparib) or 10 μmol/L to 64 nmol/L (M4344) in complete organoid medium. A 7 × 7 dose–response matrix was constructed, and each drug was also used alone to generate reference curves for each individual compound. DMSO at a concentration of 0.3% was included as a negative control (mock) for normalization purposes. Topotecan at a concentration of 1 μmol/L was included as a positive control and to evaluate the quality of the assay. All treatments were prepared at 10× concentration, and 4.4 μL of each mixture was added to the initial 40 μL of organoid culture in the wells. Three technical replicates were used in each experiment. All plates were imaged by brightfield acquisition every 24 hours for 7 days using Incucyte SX1 (Sartorius) to monitor the PDX-O responses to treatments. To visualize the live/dead nucleated cells in PDX-Os, the dual-fluorescence Cyto3D Live-Dead assay (#BM01, Tebubio) was applied at 1% v/v in each well, following the manufacturer’s recommendations. Dual-fluorescence viability signal and bright-field images were acquired using Incucyte SX1 (Sartorius), and correlative measures of cell viability were subsequently obtained by using CellTiter-Glo 3D (#G9682, Promega) on the same wells after 7 days, following the manufacturer’s instructions.

### Immunofluorescence and image analysis

For the detection of γH2AX and RAD51 foci and micronuclei, cells were seeded in black 96-well plates (Greiner Bio-One, #655090) at a density of 12,000 cells per well and exposed to the indicated drugs for 72 hours. Cells were then fixed in 4% paraformaldehyde for 20 minutes at room temperature, washed twice with PBS, and permeabilized with 0.5% Triton X-100 in PBS for 10 minutes. Cells were then blocked in immunofluorescence buffer (IFF; 2% BSA and 2% FBS in PBS) for 1 hour at room temperature and incubated with primary antibodies (RAD51, Abcam, ab133534; γH2AX, Millipore, 05-636; dilution 1:1,000 in IFF) at 4°C overnight. Cells were then washed twice with PBS and incubated with Alexa Fluor 488–conjugated rabbit (Thermo Fisher Scientific, A-11008, dilution 1:1,000) or Alexa Fluor 647–conjugated mouse secondary (Thermo Fisher Scientific, A-21235, dilution 1:1,000) antibodies and 1 μg/mL 4',6-diamidino-2-phenylindole (DAPI). For micronuclei assessment, the cells were incubated with PicoGreen (1:400 with IFF). Cells were then washed twice with PBS, and 100 μL PBS was added to each well prior to imaging. Plates were imaged using an ImageXpress Micro Confocal High-Content Imaging System (Molecular Devices). Nine independent and randomly selected sites were scanned per well. Quantification of the number of γH2AX foci, RAD51 foci, and micronuclei was performed under identical microscopy settings between the samples, using the MetaXpress image analysis system (Molecular Devices).

### DNA fiber combing

JN1 cells were grown in 100-mm dishes and synchronized using a double-thymidine block. Synchronized cells were transfected with EWS–WT1 or CCND1 siRNAs as described above. After 8 hours, cells were continuously exposed to either DMSO control, talazoparib, M4344, or a combination of both for 6 hours. For replication fork labeling, cells received prewarmed medium containing 100 μmol/L 5-chloro-2′-deoxyuridine and were incubated at 37°C and 5% CO_2_ for 30 minutes. Cells were then rinsed three times with prechilled PBS and incubated with 100 μmol/L iodo-deoxyuridine for 30 minutes. Cells were collected in cold PBS, counted, and adjusted to 50,000 cells per 50 μL PBS on ice. Plugs were generated by adding 50 μL of prewarmed 1% low–melting point agarose to the cells. The resulting 100 μL mix was gently homogenized and quickly transferred into a casting mold and incubated for 1 hour at 4°C to solidify. Subsequent steps were performed as previously described (14). For the analysis, initiation, termination, and cluster patterns of replicative forks were considered to measure fork velocity.

### Statistical analyses

Apart from the mouse xenograft experiment, no statistical methods were used to predetermine sample size, and experiments were not randomized. The investigators were not blinded during xenograft experiments. Unless otherwise stated, all graphs show mean values with error bars (SD); 95% confidence intervals were used and considered significance at *, *P* < 0.05; **, *P* < 0.01; ***, *P* < 0.001; ****, *P* < 0.0001; ns, not significant.

### Data availability

The raw data generated in this study are available upon request from the corresponding authors. The high-throughput drug screens analyzed in this study are publicly available and accessible in DepMap Repurposing Public 23Q2 at https://figshare.com/articles/dataset/Repurposing_Public_23Q2/23600310 or in the CellMinerCDB database at https://discover.nci.nih.gov/rsconnect/SarcomaCellMinerCDB/. Publicly available RNA sequencing (RNA-seq) data analyzed in this study were obtained from Gene Expression Omnibus with accession number GSE263523. Additional method details are available in Supplementary information. All uncropped images of the blots included in this study are also available in Supplementary information.

## Results

### Small-molecule inhibitor screening identifies PARP and ATR as targetable vulnerabilities in DSRCT

**Figure 1.**
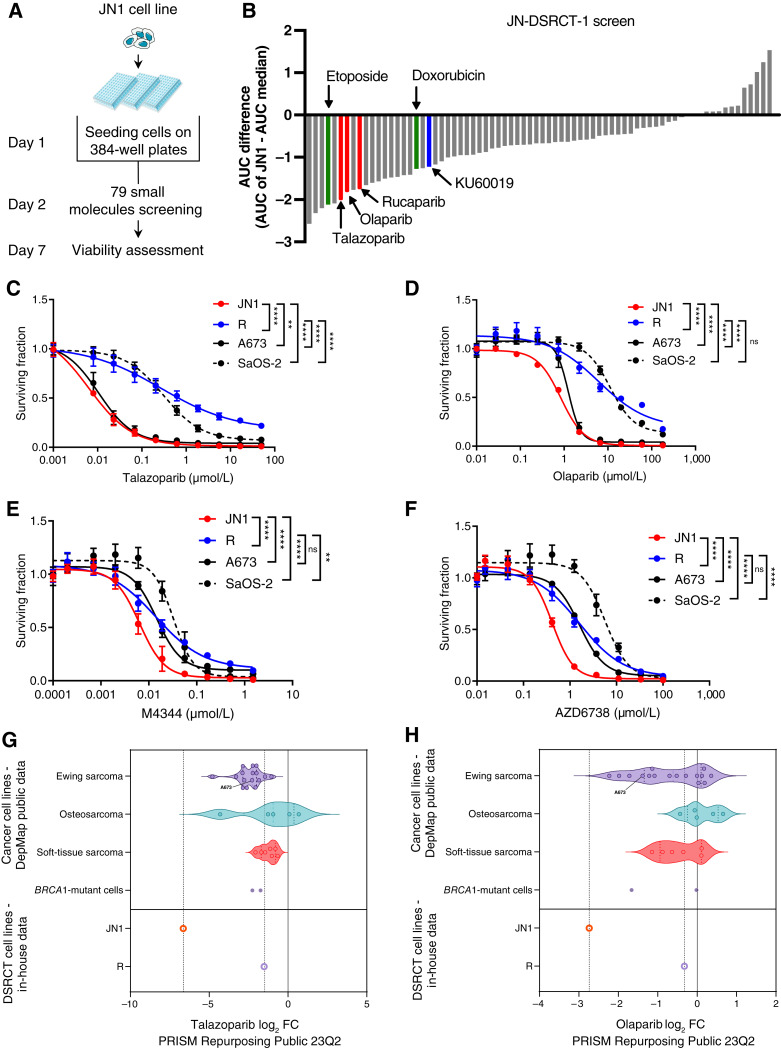
A small-molecule inhibitor and drug screen identifies PARPi and ATRi as candidate therapies for DSRCT. **A,** Schematic illustration of the workflow of small-molecule inhibitor and drug screen performed on the JN1 cell line. **B,** Waterfall plot displaying the difference in AUC between the JN1 cell line (AUC_JN1_) and the panel of 92 cell lines used for comparison (AUC_median_) for the 79 evaluated small-molecule inhibitors or drugs. Red, PARPi; blue, ATRi; green, conventional cytotoxic. **C–F,** Dose–response survival curves of the DSRCT cell lines JN1 and R, and the A673 (Ewing sarcoma) and SaOS-2 (osteosarcoma) cell lines exposed to talazoparib (**C**), olaparib (**D**), M4344 (**E**), or AZD6738 (**F**) for 7 days. Mean ± SD; *n* = 3. **G** and **H,** Violin plots showing the relative sensitivity (log_2_-fold change of cell viability) of cell lines exposed to the PARPi talazoparib (**G**) or olaparib (**H**) after a single-dose exposure at 2.5 μmol/L for 5 days in the DepMap database (PRISM Repurposing 23Q2), in comparison with that of the JN1 and R cell lines. JN1 and R cell line sensitivities were extrapolated from the survival assays presented in **C** and **E**; SFs were calculated at 2.5 μmol/L and log_2_ transformed. Ewing sarcoma cell lines (*n* = 16): RDES, A673, SKES1, CADOES1, EWS502, MHHES1, EW8, A673STAG2KO16, A673STAG2KO45, A673STAG2NT14, A673STAG2NT23, CBAGPN, CHLA10, SKNEP1, SKPNDW, and TC32; osteosarcoma cell lines (*n* = 5): G292CLONEA141B1, MG63, U2OS, HOS, and SJSA1; soft-tissue sarcoma cell lines (*n* = 7): S117, TE617T, HT1080, HS729, RD, RKN, and RH30, including rhabdomyosarcoma (*n* = 4), leiomyosarcoma (*n* = 1), fibrosarcoma (*n* = 1), and NOS sarcoma cell lines (*n* = 1), respectively. The *BRCA1/2*-mutant IGROV1 ovarian cancer cell line and *BRCA1*-mutant MDA-MB-436 breast cancer cell line were used as positive controls for sensitivity to PARPi. **, *P* < 0.01; ****, *P* < 0.0001; ns, not significant.

To identify candidate therapeutic targets for DSRCT, we conducted a high-throughput small-molecule inhibitor sensitivity screen in the JN1 cell line, using an in-house curated library of 79 antitumor agents and small-molecule inhibitors that are either in clinical use or in late-stage clinical development (Fig. 1A and B; Supplementary Table S1; ref. 12). We calculated normalized AUC *Z*-scores from dose–response survival curves of each drug in the JN1 cell line (Supplementary Table S3) and compared them with those of a panel of 92 tumor cell lines previously screened with the same library (Supplementary Table S4; ref. 12). This identified several DNA repair inhibitors as being highly toxic to the JN1 cell line, including three clinical PARPi (talazoparib, olaparib, and rucaparib, ranked #6, #7, and #9, with *Z*-scores of −2.018, −1.828, and −1.762, respectively) and one ataxia telangiectasia mutated inhibitor (ATMi)/ATRi (KU60019, ranked #18; *Z*-score, −1.2225). Several conventional cytotoxic agents that are in clinical use for the treatment of DSRCT were also identified, such as etoposide and doxorubicin (ranked #4 and #18, respectively; Fig. 1B).

Because PARPi are already approved in solid tumors and are evaluated in combination with ATRi in multiple clinical trials, including in pediatric populations (15), these small-molecule inhibitor classes harbored a high potential for immediate clinical translatability, and we selected them for further validation. We conducted validation experiments using several clinical PARPi and ATRi in two DSRCT cell lines: the JN1 cell line and a novel cell line, named “R,” which we created from a PDX (gift from Champions Oncology). The A673 (Ewing sarcoma) and SaOS-2 (osteosarcoma) cell lines were used comparatively as a sensitive and resistant control sarcoma model, respectively, based on publicly available PARPi and ATRi sensitivity datasets [Genomics of Drug Sensitivity in Cancer (GDSC) database and Holme and colleagues (12)]. Dose–response survival assays confirmed the sensitivity of JN1 cells to two clinical-grade PARPi (talazoparib and olaparib) and two clinical-grade ATRi (AZD6738 and M4344), with SF_50_ values similar to that of the PARPi-sensitive A673 cell line (Fig. 1C–F; JN1 vs. A673: talazoparib, *P* = 0.0095; olaparib, *P* < 0.0001; AZD6738, *P* < 0.0001; M4344, *P* < 0.0001; two-way ANOVA). When comparing the SF_50_ of PARPi and ATRi found in the JN1 cell line with the corresponding average steady-state or max single-dose plasma concentrations (*C*_ss-mean_ or *C*_sd-max_, respectively) dosed in patients enrolled in pharmacokinetic studies and treated at the recommended phase II dose (16–18), we observed that the concentrations that we used *in vitro* seemed clinically achievable (talazoparib, SF_50_ ≃ 10 nmol/L, *C*_ss-mean_ = 7 nmol/L; olaparib, SF_50_ ≃ 1 μmol/L, *C*_ss-mean_ = 1.7 μmol/L; AZD6738, SF_50_ ≃ 0.5 μmol/L, *C*_sd-max_ = 4.5 μmol/L; M4344, SF_50_ ≃ 7 nmol/L, *C*_sd-max_ = 750 nmol/L)—although no robust conclusion could be drawn at this stage considering the difficulties in comparing *in vitro* data with exposure in patients. We therefore further compared sensitivity to talazoparib or olaparib of the JN1 cell line with that of other sarcoma cell lines in publicly available datasets (DepMap, Broad Institute, Fig. 1G and H; Sarcoma CellMinerCDB (19), Supplementary Fig. S3A and S3B) and found that JN1 was as sensitive to PARPi olaparib and talazoparib as other Ewing sarcoma cell lines, consistent with their previously reported sensitivity to PARPi (20).

R cells also showed sensitivity to ATRi but were resistant to PARPi, with an SF_50_ similar to that of the PARPi-resistant SaOS-2 cell line (Fig. 1C–F; R vs. SaOS-2: talazoparib, *P* < 0.0001; olaparib, ns; AZD6738, *P* < 0.0001; M4344, *P* < 0.0004; two-way ANOVA). This prompted us to explore the known causes of primary resistance to PARPi, such as the loss of PARP1 expression, which abrogates PARP1 trapping–mediated cytotoxicity of PARPi (21). To test this hypothesis, we first evaluated PARP1 protein expression levels in JN1 and R cells by Western blotting and found that R cells displayed a significantly lower expression of PARP1 than JN1 cells (Supplementary Fig. S4A). To further establish a causative link between PARP1 expression and sensitivity to PARPi in DSRCT cells, we then evaluated the effects of silencing *PARP1* on the sensitivity of JN1 and R cells to PARPi. siRNA-mediated knockdown of *PARP1* conferred resistance to PARPi in JN1 but not R cells (Supplementary Fig. S4B–S4E). In addition, we noted that *PARP1* silencing did not affect the sensitivity of JN1 cells to veliparib—a PARPi with limited ability to trap PARP1 despite its ability to inhibit PARylation (Supplementary Fig. S4F and S4G; refs. 22, 23). Together, these findings suggest that PARP1 expression is a determinant of PARPi sensitivity in DSRCT cell lines and that PARP1 trapping contributes to the cytotoxic effect of PARPi in DSRCT.

**Figure 2.**
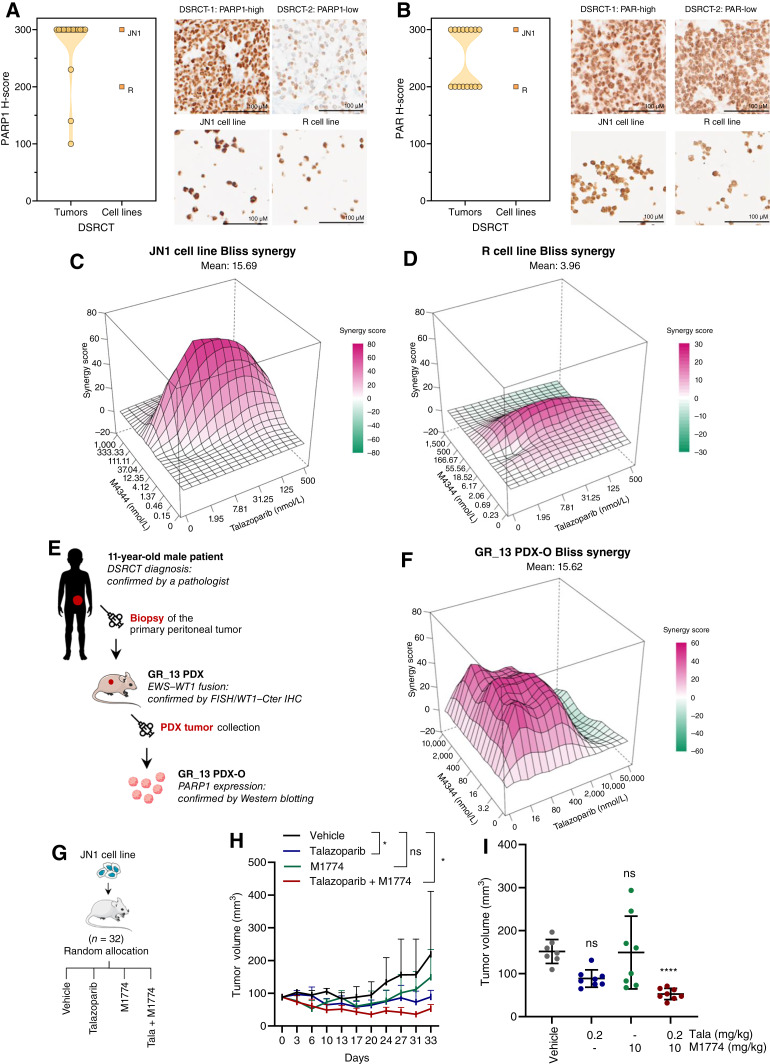
PARPi and ATRi have synergistic cytotoxic effects in models of DSRCT with high PARP1 expression. **A** and **B,** PARP1 expression (**A**) and PARylation levels (**B**) as assessed by IHC in a cohort of 16 DSRCT samples, compared with those of the JN1 and R cell lines (PARP1 and PAR expression levels are shown as H-scores). Representative cases (PARP1-high vs. PARP1-low tumors; PAR-high vs. PAR-low tumors) are shown to the right, compared with JN1 and R cells. **C** and **D,** Surface plots of Bliss independence scores calculated for the talazoparib–M4344 combination in JN1 (**C**) and R (**D**) cell lines at 7 days. **E,** The GR_13-PDX-O model was established from the primary peritoneal tumor of a patient with DSRCT, with confirmation of *EWSR1::WT1* fusion by FISH and WT1-Cter IHC (Supplementary Fig. S8). **F,** Surface plot of Bliss independence scores calculated for the talazoparib–M4344 combination in the GR_13 PDX-O at 7 days. Mean ± SD; *n* = 3. Surface plots: the *x*-axis and *y*-axis values indicate drug concentrations, and the *z*-axis values indicate the associated synergy score; score < −10, antagonistic interaction; score = 0, absence of interaction; score > 10, synergistic interaction. **G,** Schematic illustration of an *in vivo* therapeutic experiment performed to evaluate the antitumor effect of PARPi talazoparib and ATRi M1774 in NSG mice engrafted with JN1 xenografts. **H,** Therapeutic responses to drug treatment in mice harboring JN1 xenografts. Mean tumor volume ± SD; two-way ANOVA and *post hoc* Dunnett test. **I,** Tumor volume at the time of mice sacrifice. Mean ± SD; one-way ANOVA and *post hoc* Šídák test. *, *P* < 0.01; ns, not significant. Tala, talazoparib.

To next explore the applicability of our findings to patients’ tumors, we analyzed (i) PARP1 expression by RNA-seq (29 samples; ref. 24); (ii) PARP1 expression by IHC (16 samples); and (iii) PARylation levels by IHC (i.e., levels of poly (ADP-ribose), the product of PARP1 activity; 16 samples) in two DSRCT cohorts. This revealed that PARP1 was highly expressed in a large majority of the cases [24 of 29 samples (82.8%) with PARP1 expression >10 transcripts per million (TPM) by RNA-seq and 14 of 16 samples (87.5%) with PARP1 H-score ≥ 200 by IHC; Fig. 2A; Supplementary Fig. S5A] and was active (all samples with PARylation H-score ≥ 200 by IHC; Fig. 2B) as previously reported (20). As PARPi are mostly toxic by trapping PARP1 onto the DNA, we assumed that our conclusions may be applicable to most DSRCT. We further noted that the patients whose tumors harbored higher PARP1 transcript levels tended to have a longer overall survival, although this did not reach significance (Supplementary Fig. S5B).

### Combination of PARPi and ATRi shows synergistic effects in preclinical models that express PARP1

Because several PARPi plus ATRi combinations are currently being investigated in early-phase clinical trials (e.g., NCT04972110 and NCT03462342), we evaluated this combination in DSRCT cell lines. Synergy scores calculated according to the Bliss independence method showed a synergistic interaction of talazoparib plus M4344 combination in JN1 (Bliss synergy score = 15.69; Fig. 2C; Supplementary Fig. S6A) but not in R cells—in which a modest additive effect could be observed, consistent with the limited sensitivity of the latter cell line to PARPi monotherapy (Bliss synergy score = 3.96; Fig. 2D; Supplementary Fig. S6B). As CHK1i, which control the same cell-cycle checkpoint and signaling pathway as ATRi, have also been evaluated in DSRCT in combination with irinotecan (NCT04095221), we further evaluated the combination of PARPi with CHK1i. In our original screen, the evaluated CHK1i displayed limited cytotoxic effects in monotherapy in the JN1 cell line (SAR-20106, rank #39, *Z*-score = −0.697; PF-00477736, rank #55, *Z*-score = −0.197). We therefore used the clinical-grade CHK1i prexasertib and found additive effects with talazoparib in the JN1 cell line (Bliss synergy score = 6.00) but not in the R cell line, again consistent with the limited sensitivity of the latter to PARPi monotherapy (Supplementary Fig. S6C–S6F). As PARPi and irinotecan—which is part of the chemotherapy regimen for patients who suffer from DSRCT—have some partly overlapping mechanism of action through DNA double-strand break formation, we assessed the combination of SN-38 (the active metabolite of irinotecan) with two clinical compounds that target the G_2_–M cell-cycle checkpoint: the ATRi M6620 and the CHK1i SRA-737. Using a 3D spheroid model derived from the JN1 cell line that represents DSRCT cell physiology better than two-dimensional (2D) cultures, we found that both inhibitors enhanced the cytotoxic effects of irinotecan—with M6620 showing potentially the most prolonged antiproliferative potential (Supplementary Fig. S7). The concentrations of SN-38, M6620, and SRA-737 evaluated in these assays were lower than that clinically achievable in patients based on the *C*_sd-max_ described for these compounds [SN-38, *C*_sd-max_ = 33 nmol/L (25); SRA-737, *C*_sd-max_ = 1.440 μmol/L (26); and M6620, *C*_sd-max_ = 740 nmol/L (27)], altogether supporting the relevance of our observations made with PARPi.

To further confirm the sensitivity of DSRCT to PARPi and ATRi, we sought to use a third, independent, biologically distinct, and clinically relevant model. As 3D and patient-derived models reportedly better recapitulate the clinical reality than do 2D cultures or established cell line models (28–30), we sought to develop a new primary patient-derived organoid model of DSRCT. To do so, we first established a PDX model from the primary peritoneal tumor of an 11-year-old male patient with DSRCT and subsequently created a PDX-O, referred to as GR_13, in which we assessed the sensitivity to PARPi, ATRi, and the combination of both agents (Fig. 2E; Supplementary Fig. S8). These experiments revealed cytotoxic effects of PARPi and ATRi monotherapies against GR_13 PDX-Os and confirmed the synergistic effects of their combination (Bliss independence score = 15.62; Fig. 2F; Supplementary Fig. S9A–S9C), albeit at high concentrations, in line with the known heightened drug resistance of 3D models when compared with 2D models (31). In line with PARP1 expression confirmed by Western blotting in GR_13 PDX-O (Supplementary Fig. S8C) and previous findings in the JN1 cell line (Fig. 2C; Supplementary Fig. S6A), this result confirmed our previous observations and the sensitivity of DSRCT to PARPi plus ATRi combinatorial strategy.

We next assessed the therapeutic potential of an ATRi plus PARPi combination *in vivo* and evaluated the antitumor effect of PARPi talazoparib, ATRi M1774, or a combination of both agents in mice bearing established xenografts from the JN1 cell line (Fig. 2G). As JN1 tumors do not grow in nude mice, we used NSG mice that carry the *Prkdc*^scid^ mutation, which confers exquisite sensitivity to DNA damaging agents and chronic exposure to ATRi. This required the use of a minimally toxic schedule of drug administration for a total maximum duration of 33 days. In this experiment, we found that compared with the drug vehicle, both talazoparib and M1774 monotherapies reduced tumor growth of JN1 xenografts (Fig. 2H and I; *P* < 0.001, two-way ANOVA). The combination therapy further reduced tumor growth and caused tumor shrinkage (Fig. 2H and I; Supplementary Fig. S10A, B; median tumor volume: 151.5 mm^3^ in the vehicle arm vs. 62.6 mm^3^ in the combination arm; *P* < 0.0001, two-way ANOVA). Altogether, these results suggested that the combination of PARPi and ATRi could act synergistically in DSRCT cells that express PARP1, both *in vitro* and *in vivo*.

### Combination of PARPi and ATRi elicits DNA damage, replication stress, and genomic instability in DSRCT cells

**Figure 3.**
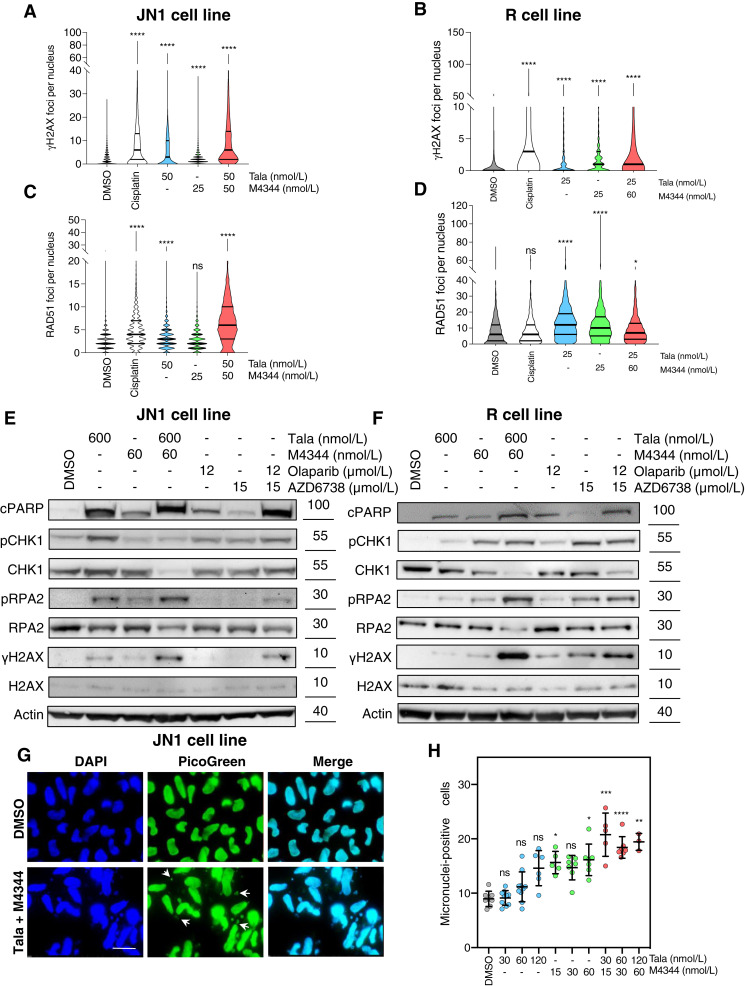
PARPi and ATRi combination elicits DNA damage, replication stress, and genomic instability in DSRCT cells. **A–D,** Quantification of γH2AX (**A** and **B**) or RAD51 foci (**C** and **D**) in JN1 (**A** and **C**) or R (**B** and **D**) cells exposed to DMSO control, PARPi talazoparib (Tala), ATRi M4344, or a combination of both for 72 hours. Cisplatin was used as the positive control. A minimum of 500 nuclei was analyzed per condition. Violin plots show the absolute number of foci per nucleus. Thick line, median; thin lines, bottom and top quartiles; two-way ANOVA and *post hoc* Dunn test. **E** and **F,** Western blots of pCHK1, CHK1, pRPA2, RPA2, γH2AX, H2AX, and cleaved-PARP1 (cPARP) in JN1 (**E**) or R (**F**) cells exposed to DMSO control, PARPi talazoparib or olaparib, ATRi M4344 or AZD6738, or a combination of both for 48 hours. **G** and **H,** Representative immunofluorescence images (**G**) and quantification (**H**) of micronuclei-positive cells in JN1 cells exposed to DMSO control, PARPi talazoparib, ATRi M4344, or a combination of both for 72 hours. A minimum of 500 cells was analyzed per condition. Mean ± SD; *n* = 3; one-way ANOVA and *post hoc* Dunn test. Arrows, micronuclei. Scale bar, 20 μm. *, *P* < 0.05; **, *P* < 0.01; ***, *P* < 0.001; ****, *P* < 0.0001; ns, not significant.

To understand the molecular mechanisms underlying this vulnerability in DSRCT cells, we first sought to explore the known causes of PARPi and ATRi sensitivity and assessed DNA damage, homologous recombination (HR) functionality, and replication stress. We found that exposure to PARPi and ATRi led to increased DNA damage, as assessed by immunofluorescence detection of γH2AX foci in JN1 and R cells (Fig. 3A and B). This effect was concentration dependent (Supplementary Fig. S11A and S11B) and significantly enhanced in the context of PARPi plus ATRi combination using several clinical-grade agents, with γH2AX foci levels being similar to those induced by cisplatin (Fig. 3A and B). We further noted that γH2AX foci accumulation was (i) overall more pronounced in JN1 cells than in R cells exposed to the combination therapy and (ii) limited in R cells exposed to PARPi as a monotherapy, consistent with the low PARP1 expression and limited PARPi sensitivity of this cell line. We next assessed HR function by quantifying the levels of RAD51 foci and found that these were significantly increased in response to PARPi (Fig. 3C and D; Supplementary Fig. S11C and S11D) but not to ATRi monotherapy, in line with the current literature suggesting that ATR promotes RAD51 accumulation at double-strand breaks (32). This effect was enhanced when both agents were combined, to a higher extent than cisplatin exposure (Fig. 3C and D). Altogether, these results suggested that DSRCT cells are HR proficient and that their sensitivity to PARPi and ATRi does not result from a HR defect.

By mediating PARP1 trapping onto DNA, PARPi are known to increase reliance upon the ATR/CHK1 pathway because of increased stalled replication forks and resultant replication stress (33). ATR is a master regulator of the DNA damage response, which coordinates cell-cycle transitions with the DNA replication, DNA repair, and apoptotic machineries to prevent the deleterious effects of replication stress. ATR activation leads to phosphorylation of CHK1 (pCHK1) and other ATR effectors, which ultimately slows down origin firing, induces cell-cycle arrest in response to DNA damage, and promotes stabilization and restart of stalled replication forks (34, 35). We evaluated, by using Western blotting, the phosphorylation of ATR and CHK1 and found increased pCHK1 levels upon PARPi and ATRi exposure in JN1 and R cells (Fig. 3E and F; Supplementary Fig. S11E), suggesting an activation of the replication stress checkpoint. To further investigate the presence of ongoing replication stress, we evaluated the levels of RPA2 phosphorylation (pRPA2) and found increased pRPA2 levels upon exposure to PARPi, ATRi, and their combination. This was associated with increased DNA damage and apoptosis (as assessed by γH2AX and PARP1 cleavage, respectively; Fig. 3E and F; Supplementary Fig. S11E), consistent with our previous observations (Fig. 3A and B; Supplementary Fig. S11A and S11B).

To further assess the genomic consequences of PARPi plus ATRi combination in DSRCT, we measured levels of micronuclei—cytoplasmic chromosome fragments that arise during mitosis from lagging chromosomal DNA or chromatin bridges as a result of unresolved DNA lesions. We found that the combination of PARPi and ATRi significantly increased the number of micronuclei in JN1 cells compared with the DMSO control or either of the corresponding monotherapies (Fig. 3G and H). A similar effect was observed in R cells (Supplementary Fig. S11F), although to a lesser extent, in line with their lower levels of PARP1 expression. Altogether, these findings indicate that combined exposure to PARPi and ATRi elicits high levels of DNA damage, replication stress, and micronuclei in DSRCT cells, in a context of functional HR repair.

### EWS–WT1 is a determinant of sensitivity to PARPi and ATRi in DSRCT

We next sought to explore whether the EWS–WT1 chimeric transcription factor was the cause of PARPi and ATRi sensitivity in DSRCT cells. Indeed, although the *EWSR1::WT1* gene fusion is the known driver of DSRCT, it remained possible that other alterations in DSRCT cells could cause the drug sensitivity effects seen. For example, the t(11;22)(q13;q12) chromosomal translocation, beyond causing *EWS*–*WT1* fusion, also alters the chromosomal location of genes that flank either *EWSR1* or *WT1*.

**Figure 4.**
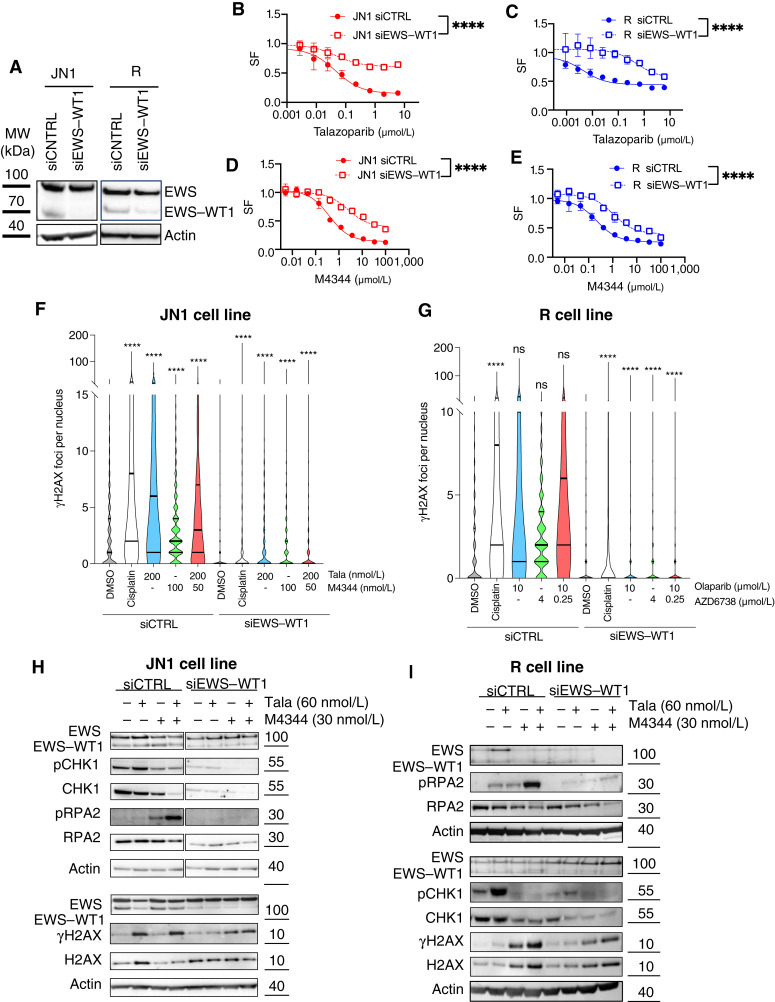
EWS–WT1 is a determinant of DSRCT cells’ sensitivity to PARPi and ATRi. **A,** Western blot of EWS–WT1 in JN1 and R cells transfected with either siCNTRL or siEWS–WT1. Whole-cell lysates were generated 48 hours after transfection. **B–E,** Dose–response survival curves of JN1 or R cells exposed to PARPi talazoparib (**B** and **C**) or ATRi M4344 (**D** and **E**) for 7 days in the presence or absence of siRNA-mediated silencing of EWS–WT1. Mean ± SD; *n* = 3. **F** and **G,** Quantification of γH2AX in JN1 cells exposed to DMSO control, PARPi talazoparib, ATRi M4344, or a combination of both for 72 hours, in the presence or absence of siRNA-mediated silencing of EWS–WT1. Cisplatin was used as the positive control. A minimum of 500 nuclei was analyzed per condition. Violin plots show the absolute number of foci per nucleus. Thick line, median; thin lines, bottom and top quartiles; two-way ANOVA and *post hoc* Dunn test. **H** and **I,** Western blots of pCHK1, CHK1, pRPA2, RPA2, γH2AX, H2AX, and EWS–WT1 in JN1 (**H**) or R (**I**) cells exposed to DMSO control, PARPi talazoparib (Tala), ATRi M4344, or a combination of both for 48 hours, in the presence or absence of siRNA-mediated silencing of EWS–WT1. ****, *P* < 0.0001; ns, not significant.

To do so, we designed siRNAs targeting the specific breakpoints of the *EWS–WT1* fusion in the JN1 and R cell lines, respectively (Fig. 4A; Supplementary Figs. S1 and S2) and explored the effect of *EWS*–*WT1* silencing on the above-described phenotypes. We first assessed cell survival upon PARPi or ATRi exposure and observed that *EWS*–*WT1* silencing conferred increased resistance to both agent classes (Fig. 4B–E; Supplementary Fig. S12A–S12D), suggesting the existence of a common EWS–WT1–dependent mechanism driving sensitivity to both agents. Of note, silencing of *CCND1*—a direct target of EWS–WT1 (36)—conferred little increased resistance to PARPi or ATRi compared with *EWS*–*WT1* silencing in the JN1 cell line (Supplementary Figs. S13 and S14A–S14D), suggesting that the sensitivity to PARPi and ATRi induced by the fusion was, at least in part, independent of the role of EWS–WT1 in modulating *CCND1*. Similarly, and in line with this hypothesis, CDK1i-mediated cell-cycle blockade failed to phenocopy the effects of siRNA-mediated *EWS*–*WT1* silencing toward increasing the resistance of DSRCT cells to either PARPi or ATRi (Supplementary Fig. S14E and S14F). This overall suggested that the sensitivity to PARPi and ATRi induced by the fusion was, at least in part, independent of its role in modulating *CCND1* expression and the cell-cycle profile (Supplementary Fig. S13). We further found that levels of DNA damage induced by PARPi and ATRi were significantly reduced upon *EWS–WT1* silencing, as assessed by immunofluorescence detection of γH2AX foci (Fig. 4F and G). To confirm the role of EWS–WT1 in PARPi- and ATRi-mediated effects, we next assessed ATR/CHK1 pathway activity by Western blotting and found that (i) the PARPi-induced pCHK1 response was abrogated upon *EWS*–*WT1* silencing and (ii) the pRPA2 and γH2AX responses elicited by PARPi plus ATRi combination were either reversed or significantly attenuated upon *EWS*–*WT1* silencing (Fig. 4H and I). These findings suggested that *EWS*–*WT1* is required for the sensitivity of DSRCT cells to PARPi, ATRi, and their combination.

### EWS–WT1 increases endogenous DNA replication stress and R-loops, which drive sensitivity to PARPi and ATRi

Because we observed that the sensitivity to PARPi and ATRi was EWS–WT1 dependent and oncogenic transcription factors have been reported to increase replication stress (37), we next focused on replication forks and their functionality.

**Figure 5.**
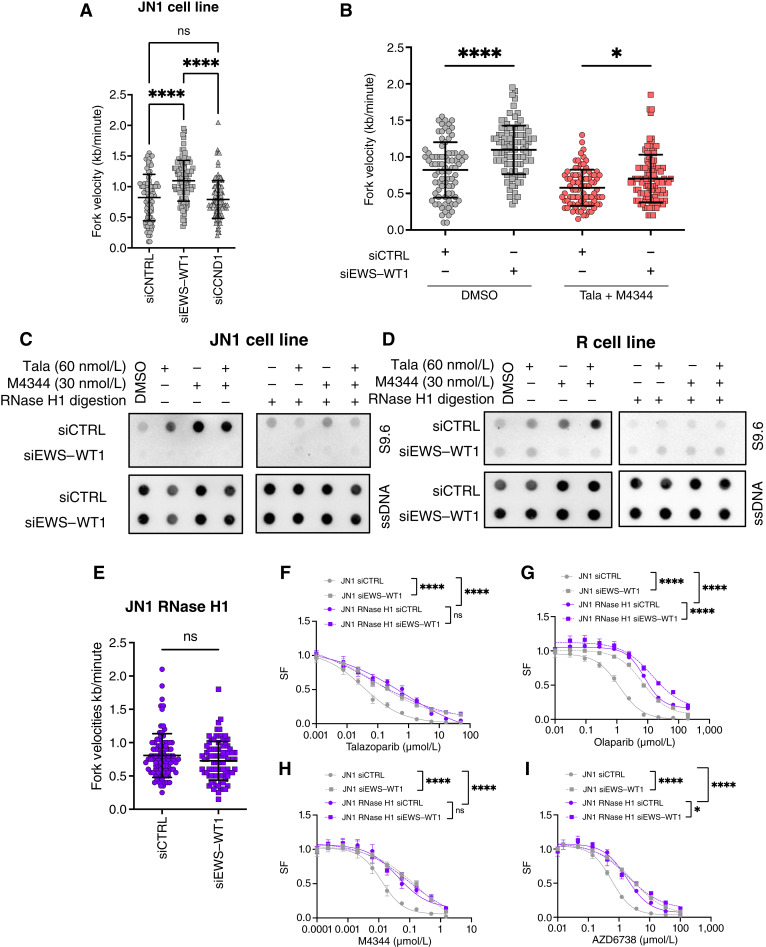
EWS–WT1 drives enhanced DNA replication stress and R-loops, which contribute to DSRCT cells’ sensitivity to PARPi and ATRi. **A,** Assessment of replication fork speed (kb/minute) in JN1 cells subjected to siRNA-mediated silencing of EWS–WT1 or CCND1. A minimum of 50 forks was analyzed per condition. Mean ± SD; each dot represents a single replication fork; *n* = 2, one-way ANOVA and *post hoc* Dunnett test. **B,** Assessment of replication fork speed (kb/minute) in JN1 cells exposed to DMSO control, or a combination of PARPi talazoparib (Tala) and ATRi M4344 for 6 hours, in the presence or absence of siRNA-mediated silencing of EWS–WT1. A minimum of 50 forks was analyzed per condition. Mean ± SD; each dot represents a single replication fork; *n* = 2; two-way ANOVA and *post hoc* Šídák test. **C** and **D,** DNA:RNA hybrid dot blot of genomic DNA extracted from JN1 (**C**) or R (**D**) cells exposed to PARPi talazoparib, ATRi M4344, or a combination of both in the presence or absence of siRNA-mediated silencing of EWS–WT1 as in **B**. S9.6, RNA:DNA hybrids; ssDNA, loading control. **E,** Assessment of replication fork speed (kb/minute) in RNase H1–overexpressing JN1 cells subjected to siRNA-mediated silencing of EWS–WT1. Synchronized cells were collected 14 hours after transfection. A minimum of 50 forks was analyzed per condition. Mean ± SD; each dot represents a single replication fork; *n* = 2; unpaired *t* test. **E,** Dose–response survival curves of JN1 cells exposed to PARPi talazoparib (**F**) or olaparib (**G**), and ATRi M4344 (**H**) or AZD6738 (**I**) for 7 days in the presence or absence of siRNA-mediated silencing of EWS–WT1 and/or RNase H1 overexpression. Mean ± SD; *n* = 3; two-way ANOVA. *, *P* < 0.05; ****, *P* < 0.0001; ns, not significant.

We first investigated replication fork progression upon silencing of *EWS*–*WT1* using the DNA fiber combing assay in the JN1 cell line. We found that *EWS*–*WT1* silencing caused >30% increase in fork velocity (siCNTRL, 0.82 kb/minute vs. siEWS–WT1, 1.1 kb/minute; *P* < 0.0002, Mann–Whitney *U* test; Fig. 5A) in the absence of drug exposure. Interestingly, this effect was not observed upon *CCND1* silencing (Fig. 5A), suggesting that EWS–WT1–induced reduction in replication fork velocity was, at least in part, independent of its effects in driving cell proliferation through the cell cycle (Supplementary Fig. S13). We next assessed replication fork progression upon PARPi and ATRi exposure in the JN1 cell line and found that their combination decreased fork velocity (siCNTRL DMSO, 0.82 kb/minute vs. siCNTRL talazoparib + M4344, 0.58 kb/minute; *P* < 0.0001, Mann–Whitney *U* test; Fig. 5B; Supplementary Fig. S15A), in line with these agents’ mechanism of action and increased replication stress. This effect was partially rescued by *EWS*–*WT1* silencing (siCNTRL talazoparib + M4344, 0.7 kb/minute vs. siEWS-WT1 talazoparib + M4344, 1.1 kb/minute; *P* < 0.0001, Mann–Whitney *U* test; Fig. 5B). Altogether, these results suggested that EWS–WT1 expression in JN1 cells increases replication stress, which is further exacerbated by PARPi and ATRi exposure.

As aberrant transcription factors not only cause replication stress but also enhance transcription, we next sought to assess R-loops. R-loops are three-stranded nucleic acid structures consisting of an RNA:DNA hybrid and a displaced nonhybridized ssDNA, which form in the genome when an RNA strand invades double-stranded DNA within the chromatin. R-loops naturally occur during replication and transcription, in which they play important roles in regulating gene expression and chromatin structure. Their aberrant accumulation can also represent a threat to genomic stability, by causing increased replication stress and subsequent DNA damage (38–42).

We first assessed R-loop levels in DSRCT cells using RNA:DNA hybrid dot blotting with the S9.6 antibody on genomic DNA extracted from JN1 or R cells. We found that *EWS*–*WT1* silencing reduced endogenous R-loop levels in both JN1 and R cells (Fig. 5C and D), whereas *CCND1* silencing had no such effect (Supplementary Fig. S15B). We next compared R-loop levels in cells exposed to PARPi, ATRi, or their combination in the presence or absence of *EWS*–*WT1* silencing. This revealed a significant accumulation of RNase H–sensitive R-loops in response to the combination, which was (i) enhanced compared with either of the corresponding monotherapies and (ii) significantly attenuated in the context of *EWS*–*WT1* silencing (Fig. 5C and D). To further explore the role of R-loops in DSRCT cells, we constructed a JN1 cell line that stably expresses an exogenous cDNA encoding *RNASEH1*, the main ribonuclease responsible for R-loop degradation in humans (herein referred to as JN1-RNaseH1; Supplementary Fig. S15C). In contrast to our previous observations in the JN1 wild-type cell line (Fig. 5A), we noted that *EWS*–*WT1* silencing had no effect on replication fork velocity in JN1-RNaseH1 (Fig. 5E), suggesting that RNaseH1 overexpression might counteract the replication stress resulting from EWS–WT1–driven R-loop burden. Strikingly, dose–response survival assays of JN1 and JN1-RNaseH1 cells exposed to various PARPi or ATRi monotherapies showed that RNase H1 overexpression conferred resistance to these inhibitors, supporting a role for R-loops in driving PARPi and ATRi sensitivity in DSRCT cells (Fig. 5F–I; Supplementary Fig. S16). Furthermore, we noted that (i) the magnitude of this effect was similar to that obtained when silencing *EWS–WT1* in JN1 cells and (ii) silencing *EWS*–*WT1* conferred no further resistance to PARPi or ATRi in JN1-RNaseH1 cells (Fig. 5F–I; Supplementary Fig. S17), supporting an epistasis between *EWS–WT1* silencing and RNase H1 overexpression in driving resistance to PARPi and ATRi. Altogether, these findings show that EWS–WT1 drives R-loop formation and a resultant increased replication stress in DSRCT cells, which underlies their sensitivity to PARPi and ATRi.

### Combination of PARPi and ATRi elicits cell-intrinsic immunity in DSRCT cell lines

The cGAS–STING pathway is a component of the innate immune response; by acting as a sensor for cytosolic DNA, cGAS activates a signaling cascade involving STING trafficking and TANK-binding kinase 1 (TBK1) and IFN regulatory factor 3 (IRF3) phosphorylation, which culminates in a type I IFN response and the subsequent upregulation of IFN-stimulated genes, such as *CCL5* and *CXCL10* (43). More recently, pharmacologic manipulation of the cGAS–STING pathway has been proposed as a therapeutic strategy, notably in cancer to render tumors “immunologically hot” as a way to facilitate response to immunotherapies (44).

**Figure 6.**
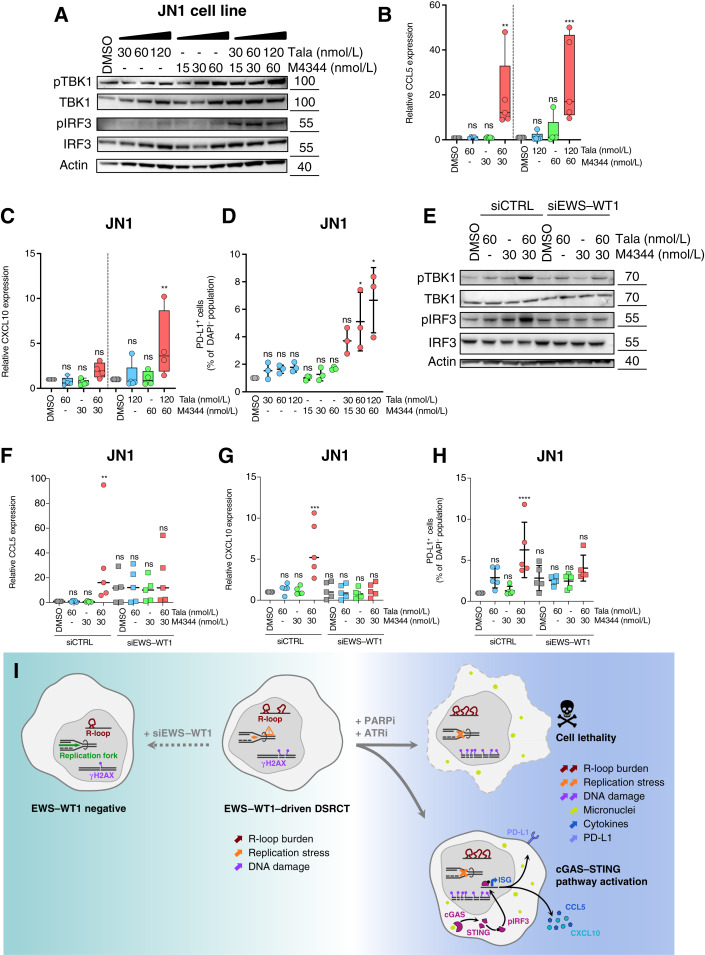
The combination of PARPi and ATRi elicits a cGAS–STING–mediated cell-autonomous immune response. **A,** Western blots of pTBK1, TBK, pIRF3, and IRF3 in JN1 cells exposed to DMSO control, PARPi talazoparib (Tala), ATRi M4344, or a combination of both for 72 hours. **B** and **C,** RT-qPCR analysis of RNA isolated from JN1 cells exposed to DMSO control, PARPi talazoparib, ATRi M4344, or a combination of both for 72 hours. CCL5 (**B**) and CXCL10 (**C**) mRNA were analyzed separately relative to RPLP0. Box and whisker plots show arbitrary units of gene expression, normalized to the DMSO condition. Boxes, median and lower and upper quartiles; whiskers, the 5th to 95th percentile range; *n* = 4; two-way ANOVA and *post hoc* Dunnett test, relative to the DMSO condition. **D,** Quantification of PD-L1 cell-surface expression by flow cytometry in JN1 cells exposed to DMSO control, PARPi talazoparib, ATRi M4344, or a combination of both for 72 hours. Scatter plot shows the percentage of PD-L1–positive cells within the DAPI-negative population, normalized to the DMSO condition. Mean ± SD; *n* = 3. Kruskal–Wallis test and *post hoc* Dunnett test, relative to the DMSO condition. **E,** Western blots of pTBK1, TBK, pIRF3, and IRF3 in JN1 cells exposed to DMSO control, PARPi talazoparib, ATRi M4344, or a combination of both for 72 hours, in the presence or absence of siRNA-mediated silencing of EWS–WT1. Appropriate silencing of EWS–WT1 was verified as shown in Fig. 4H. **F** and **G,** RT-qPCR analysis of RNA isolated from JN1 cells exposed to DMSO control, PARPi talazoparib, ATRi M4344, or a combination of both for 72 hours, in the presence or absence of siRNA-mediated silencing of EWS–WT1. CCL5 (**F**) and CXCL10 (**G**) mRNA were analyzed separately relative to RPLP0. Box and whisker plots show arbitrary units of gene expression, normalized to the siCNTRL DMSO condition. Boxes, median and lower and upper quartiles; whiskers, the 5th to 95th percentile range; *n* = 4; two-way ANOVA and *post hoc* Dunnett test, relative to the siCNTRL DMSO condition. **H,** Quantification of PD-L1 cell-surface expression by flow cytometry in JN1 cells exposed to DMSO control, PARPi talazoparib, ATRi M4344, or a combination of both for 72 hours, in the presence or absence of siRNA-mediated silencing of EWS–WT1. Scatter plot shows the percentage of PD-L1–positive cells within the DAPI-negative population, normalized to the siCNTRL DMSO condition. Mean ± SD; *n* = 3. Kruskal–Wallis test and *post hoc* Dunnett test, relative to the siCNTRL DMSO condition. **I,** Model of EWS–WT1–driven DSRCT sensitivity to PARPi and ATRi. *, *P* < 0.05; **, *P* < 0.01; ***, *P* < 0.001; ****, *P* < 0.0001; ns, not significant. ISG, IFN-stimulated genes.

Based on recent reports, including ours, describing that PARPi and ATRi can trigger a cell-autonomous type I IFN response through the activation of the cGAS–STING pathway subsequent to micronuclei formation (41, 45–50), we decided to explore the ability of PARPi and ATRi to elicit such a response in DSRCT cells. We first observed a concentration-dependent increase in TBK1 and IRF3 phosphorylation upon PARPi and ATRi exposure in JN1 cells—an effect that was enhanced in the context of their combination (Fig. 6A). We next assessed downstream CCL5 and CXCL10 expression levels by RT-qPCR and found that these chemokines were increased by more than 20-fold and 5-fold, respectively (Fig. 6B and C) upon combination therapy. This was further accompanied by a concentration-dependent increase in PD-L1 cell-surface expression, as assessed by flow cytometry (Fig. 6D). Together with our previous observation that PARPi plus ATRi combination induces micronuclei formation (Fig. 3G and H; Supplementary Fig. S11F), these data suggest that a cell-autonomous cGAS–STING–mediated type I IFN response is activated in DSRCT cells as a result of PARPi and ATRi exposure. We next investigated the role of EWS–WT1 in such a response and found that *EWS*–*WT1* silencing attenuated all of the above phenotypes, including TBK1 and IRF3 phosphorylation (Fig. 6E), CCL5 and CXCL10 upregulation (Fig. 6F–G), and PD-L1 cell-surface expression (Fig. 6H). Altogether, these results indicate that PARPi and ATRi elicit a type I IFN response in DSRCT cells that is dependent upon EWS–WT1 expression.

## Discussion

DSRCT is an extremely aggressive malignancy with very limited therapeutic options. Here, we show that preclinical models of DSRCT are selectively sensitive to clinical PARPi and ATRi. The use of functional genomics allowed us to propose a model whereby these genetic vulnerabilities are mediated by increased EWS–WT1–dependent replication stress and R-loop formation, which results in cGAS–STING pathway activation and a cell-autonomous type I IFN response (Fig. 6I), opening new therapeutic avenues to increase immunogenicity of this genetically simple, immune-cold disease.

To the best of our knowledge, our work represents the first report of the selective sensitivity of DSRCT cells to ATRi and of the involvement of EWS–WT1–dependent R-loop burden in this vulnerability. Our work specifically underlines the translational potential of combining PARPi and ATRi in DSRCT, a combination that is currently being evaluated by multiple clinical trials, including in children (NCT02813135). Previous literature has suggested sensitivity of DSRCT to PARPi in combination with the alkylating agent temozolomide, subsequent to observation of high levels of PARP1 and SLFN11 expression in DSRCT (20). Our analysis of 29 and 16 tumor samples by RNA-seq and IHC, respectively, confirms these findings, thereby reinforcing the potential of using such DNA damage response inhibitors in the treatment of patients with DSRCT. Still, our observation that some tumors do not express PARP1—a major mechanism of resistance to PARPi (21, 51)—highlights the need for careful molecular selection and verification of adequate PARP1 expression prior to treatment orientation.

The potential for using CHK1i, such as prexasertib, has also been reported in preclinical models of DSRCT (52) and further evaluated in a clinical trial in combination with irinotecan (NCT04095221; ref. 53). In the latter, 6/19 (32%) and 9/19 (47%) patients showed partial response and stable disease as the best response, respectively. The trial met its primary endpoint, supporting further investigation of this combination. Data on the PARPi plus ATRi combination in DSRCT are much scarcer for now: one heavily pretreated patient, who received the PARPi olaparib in combination with ATRi AZD6738 as part of the eSMART trial (NCT02813135), presented stable disease for 4 months of the study (15). Additional data from this trial are eagerly awaited to better evaluate the potential of this combination in patients with DSRCT. As CHK1i and ATRi both act on the G_2_–M cell-cycle checkpoint, we can anticipate that their mechanism of action partially overlaps. Based on available clinical data, the PARPi plus ATRi combination may have a better tolerability profile than the CHK1i plus irinotecan combination, notably with regard to fatigue and cytopenia (15, 53, 54). In the former combination, the oral administration of both drugs also represents an important difference between the two regimens, which may offer the advantage of higher flexibility in scheduling and dosage adaptations. However, it also represents a limitation for patients who have peritoneal disease, and are therefore at risk of malabsorption, occlusion, etc., and PARPi have shown disappointing efficacy in pediatric malignancies so far. In this context, we can hope that the use of last-generation potent PARP1-selective inhibitors (e.g., AZD5305) will allow the enhancement of PARPi efficacy while limiting hematologic toxicity.

DSRCT is related to the group of SRCSs, of which, Ewing sarcoma is the prototypic EWS–FLI1–driven disease. PARP1 inhibition has initially been proposed as a therapeutic strategy in Ewing sarcoma, subsequent to the identification of an interaction between PARP1 and the fusion transcripts that potentiated DNA damage (55). EWS–FLI1 was subsequently reported to increase the R-loop burden and disable BRCA1-dependent HR. Such a “BRCAness” phenotype was not observed in our study, in which we could detect adequate RAD51 foci formation in DSRCT cells exposed to PARPi. Thus far, PARPi have shown disappointing efficacy in patients with heavily pretreated Ewing sarcoma [reviewed in Pearson and colleagues (56)]. A few isolated responses have been observed, which deserve further molecular exploration to identify the clinically relevant biomarkers that drive sensitivity in this population. Based on these results, the most recent consensus expert guidelines from the multistakeholder Pediatric Strategy Forum on DNA repair (ACCELERATE and European Medicines Agency, with participation of the FDA) recommended to assess CHK1i and ATRi as a high priority and PARPi only in combination with the latter (56). The synergy observed preclinically upon combination of PARPi and ATRi in the JN1 cell line (Fig. 2C) and GR_13 PDX-O model (Fig. 2F) also supports the latter approach. Beyond SRCS, trabectedin—a cytotoxic drug used in routine sarcoma treatment and known to induce R-loops (57)—has been combined with PARPi in various soft-tissue sarcomas in the TOMAS trial (58). Unsurprisingly, these agents could not be used at full dose when combined, but activity (7/50 patients enrolled with PR) supported the evaluation of this combination in an ongoing phase II trial (NCT03838744). Although this combination is very poorly tolerated as compared with the PARPi plus ATRi combination, authors identified high PARP1 expression as well as an eight-gene signature (including DNA damage response genes such as *SLFN11*, *ATM*, and *BLM*) as predictors of better outcome on trabectedin plus PARPi (59). The latter may also be relevant to the PARPi plus ATRi combination.

We finally found that PARPi and ATRi trigger a cell-autonomous cGAS–STING/type I IFN response and PD-L1 upregulation in DSRCT cells. This immunomodulatory effect of DNA repair inhibitors could be exploited to increase immunogenicity of DSRCT cells, which are traditionally devoid of T cells in the tumor microenvironment, by attracting T cells within tumors and favoring sensitivity to anti–PD-1 therapy. Such an effect of PARPi and ATRi has been reported in other preclinical models with high replication stress [reviewed in Chabanon and colleagues (60)] and in clinical studies evaluating ATRi, notably in non–small cell lung cancer (NSCLC) and melanoma, in which they can potentiate or revert resistance to anti–PD-L1, respectively (61–63). For example, translational studies performed in the HUDSON phase II trial showed that ATRi AZD6738 could both induce inflammatory- and IFN-associated signatures and decrease exhausted CD8^+^ T cells in the blood of patients with NSCLC (62). Still, whether such effects are only observed in traditionally immunogenic diseases, such as NSCLC or melanoma, or also operate in DSRCT remains to be assessed.

We should still highlight several limitations to our observations. First, we only had access to a limited number of models. Indeed, DSRCT is an ultrarare disease (frequency < 1/1 million), and cell line models are challenging to create, probably because the desmoplastic microenvironment of this tumor type also favors cancer cell growth. In this study, we therefore created two previously unpublished models (one PDX-derived cell line and one PDX-derived organoid), which complemented the previously established JN1 cell line. Still, revalidation in additional models would ideally be required. Second, the difference in PARP1 expression between our models led to discrepant observations, notably in terms of synergistic or additive cytotoxic effects of the PARPi plus ATRi combination, in which PARP1 expression and trapping play a crucial role (21, 51). Our characterization of PARP1 expression and PARylation in patients with DSRCTs shows that PARP1 is expressed and active in the vast majority of cases, which is in line with previous independent results (20), thereby supporting clinical activity of PARPi in this patient population. Still, other determinants of PARPi and ATRi sensitivity, such as SLFN11 expression and replication stress levels, should also be considered, and the clinical applicability of our findings therefore remains unknown. Finally, we faced technical difficulties in assessing long-term efficacy of the PARPi plus ATRi combination *in vivo*, owing to systemic toxicity of ATRi in NSG mice caused by their constitutive *Prkdc*^scid^ mutation, and the impossibility to grow DSRCT xenografts in nude mice, which led us to prematurely stop our experiments. If recent clinical trial results show that PARPi and ATRi can be safely combined in adult and pediatric patients, the efficacy/toxicity profile of such a combination may have to be compared with that of other regimens, which also act on DNA damage response and replication stress [e.g., CHK1i and irinotecan combinations; NCT04095221 (52, 53)], to better define its role in therapeutic armamentarium. Despite these limitations, we believe that our study may have translational utility and clinical impact in DSRCT, a disease for which very few therapeutic options and no precision medicine approach are available.

In conclusion, our findings shed light on EWS–WT1–associated genetic vulnerabilities in DSRCT and provide a rationale for evaluating PARPi in combination with ATRi in this deadly disease. As the replication stress and R-loop dependency of this phenotype may also operate in other, more frequent, transcription factor–driven sarcomas, such as Ewing sarcoma or synovial sarcoma, we hope that this will favor the development of basket studies enrolling multiple biomarker-selected sarcomas and allow patients to access these therapies despite the rarity of their disease.

## Supplementary Material

Supplementary Data Supplementary File containing methods, figures and uncropped membranes.

Supplementary Tables S1-S4 Supplementary Tables S1-S4
