# Supplementary material for: Replication Stress Is an Actionable Genetic Vulnerability in Desmoplastic Small Round Cell Tumors
Source: Cancer Res. 2024 Oct 16;85(1):154–70. doi: 10.1158/0008-5472.CAN-23-3603 (PMC11694066; doi:10.1158/0008-5472.CAN-23-3603)

**Supplementary Information**

**Replication stress is an actionable genetic vulnerability**

**in desmoplastic small round cell tumors**

**Supplementary Material and Methods**

**Antibodies**

For immunoblotting and immunofluorescence, antibodies targeting the following epitopes were used: CCND1 (#55506), p-CHK1 (#2341), CHK1 (#2360), p-RPA2 (#83745), RPA2 (#35869), H2AX (#7631), p-TBK1 (#5483), TBK1 (#3013) from Cell Signalling Technology; PARP1 (ab191217), p-IRF3 (ab76493) and IRF3 (ab76409) from Abcam; γH2AX (05-636), ssDNA (MAB3868) and β-Actin (A1978) from Sigma Aldrich; EWS (sc-48404) and PARP1 (sc-8007) from Santa Cruz. Secondary antibodies for immunofluorescence were purchased from ThermoFisher (Anti-Mouse Alexa Fluor 647, A-21422; Anti-Rabbit Alexa Fluor 488, A-11034). The S9.6 antibody from Merck (MABE1095) was used for R-loop slot blots. For flow cytometry, the following antibodies were used: APC-conjugated anti-human CD274 (#329708) and APC-conjugated mouse IgG2b, κ isotype control (#400322) from BioLegend.

**RNAi and transfections**

CCND1 and PARP1 siRNA silencing experiments were performed using a pre-designed pool of four distinct siRNA species targeting different sequences of the target transcripts (Horizon Discovery, CCND1, L-003210-00-0005; PARP1, L-006656-03-0005). EWS-WT1 siRNA silencing experiments were performed using custom-designed siRNAs fitted to target the cell line-specific breakpoints of EWS-WT1 fusion protein; the following siRNA sequences were used: JN1 cells, 5’-GAUCUUGAUCUAGGUGAGA-3’; R cells, <5’-CUACGGGCAGCAGAGUGAG-3’> or <5’-CUGGUGGUGAGAAACCAUA-3’>. Cells were plated at 60% density and transfected by reverse transfection using Lipofectamine RNAiMAX (Invitrogen) transfection reagent. Transfection efficacy was assessed when at least 95% of cell growth was inhibited by concomitant and independent control transfection of cells with a *PLK1*-targeting siRNA (Horizon Discovery L-003290-00-0005). Validation of siRNA gene silencing was performed via western blotting from pools of concomitantly transfected cells, 48 h after transfection.

**Immunoblotting**

For western blotting, cells were lysed in RIPA lysis and extraction buffer (Thermo Fisher Scientific) supplemented with 0.5% phenylmethylsulfonyl fluoride (PMSF) and 1% Halt™ protease and phosphatase inhibitor cocktail (Thermo Fisher Scientific). Lysates were generated on ice and centrifuged 10 min at 16,900 g prior to supernatant collection. Lysates were subjected to electrophoresis using NuPAGE™ 4-12% Bis-Tris or NuPAGE™ 3-8% Tris-Acetate precast gels (Invitrogen, Carlsbad). After migration, proteins were transferred to a nitrocellulose membrane (GE Healthcare) using an iBlot2 dry gel transfer system (Thermo Fisher Scientific). 5% bovine serum albumin (BSA) in TBS buffer supplemented with 0.1% Tween 20 (TBST0.1%) was used to block the membrane, at room temperature (RT) for 1 h. Primary antibodies were diluted in 5% BSA in TBST0.1% and incubated at 4°C overnight. The next day, the membrane was washed three times with TBST0.1%, each for 10 min, followed by incubation with horseradish-peroxidase-conjugated secondary antibodies at RT for 1 h, in 5% milk in TBST0.1%. The membrane was washed again three times with TBST0.1%, and incubated with Clarity ECL substrate (Biorad). The membrane was imaged with a BioRad ChemiDoc XRS+ chemiluminescent detection system.

For RNA:DNA hybrid dot blotting, genomic DNA was extracted following a published protocol (Sanz & Chédin, *Nat Protoc*, 2019). Briefly, cells were trypsinized, washed with PBS and incubated in TE buffer containing 0.5% SDS and 62.5µg/mL proteinase K at 37°C overnight. The DNA was then purified using Phenol/Chloroform isoamyl alcohol and precipitated with 3M NaOAc pH 5.2 in 100% ethanol. The precipitated DNA was enzymatically digested overnight at 37°C using a cocktail of five restriction enzymes (EcoRI, HindIII, BsrgI, SspI, XbaI) used at 20U per reaction in CutSmart® buffer (New England Biolabs) containing 150mM spermidine. The digested DNA was then purified using Phenol/Chloroform isoamyl alcohol and precipitated with 3M NaOAc pH 5.2 in 100% ethanol, and the DNA pellet was dissolved in TE buffer. For RNase H treatment, the samples were digested overnight in 1X RNase H buffer (New England Biolabs) and RNase H (0.4 U/μl). Samples were loaded onto a Hybond® N+ hybridization membrane (RPN203B, GE Healthcare) by dot blotting using Bio-Dot® apparatus (Bio-rad), following the manufacturer’s protocol. After UV crosslinking at 1200 mJ, the membrane was blocked in 5% BSA/TBST 0.05% and incubated overnight with S9.6 antibody (1:1000). Secondary antibody blotting and membrane imaging were performed as for western blotting.

**Immunohistochemistry**

Archival samples from a cohort of 16 patients with DSRCT were used. For each sample, a single representative formalin-fixed paraffin embedded (FFPE) block was selected for the study. FFPE blocks were sectioned (3 μm thick) on a RM2245 microtome (Leica Biosystems) and placed onto adhesion slides (MM France). For PAR and WT1-Cter staining, automated immunohistochemistry was performed using a Leica BOND RX (Leica Biosystems). After deparaffinization and epitope retrieval using ER2 buffer pH9 (20 min at 100°C), the slides were incubated with primary antibodies (PAR, Sigma-Aldrich, #AM80, 1:250; WT1-Cter, Zytomed, #523-3994) for 1 h at RT. Detection was performed with the Bond Polymer Refine Detection kit (Leïca Biosystems, #DS9800). For PARP1 staining, automated immunohistochemistry was performed using a Benchmark ULTRA (Roche). After deparaffinization and epitope retrieval using CC2 buffer pH6 (44 min at 95°C), the slides were incubated with PARP1 primary antibody (ABD Serotec, #MCA 1522G, 1:1000) for 1 h at RT. Detection was performed with the UltraView Universal DAB detection kit (Roche, #760-500). The slides were mounted with glass coverslips (Labelians) and observed by means of a DM2000 microscope equipped with HC PL Fluotar 20×/0.50 and 40×/0.75 objectives and coupled to a DFC280 CCD camera (Leica Biosystems).

Pathological assessment of PARP1, PAR, and WT1-Cter was performed by a senior pathologist. PARP1 expression and PARylation levels were evaluated as a H-score (percentage of tumor cells stained multiplied by each intensity from 0 to 3+, value from 0 to 300) as previously described (Michels et al. Ann Oncol 2015).

**Fluorescence in situ hybridization (FISH)**

Tumor chunks from the GR_13 PDX were fixed in 4% PFA for 30 min and embedded in paraffin. FFPE blocks were sectioned (4 μm thick) on a RM2245 microtome (Leica Biosystems) and placed onto adhesion slides (MM France). The slides were deparaffinized in xylene and rehydrated through a graded series of ethanol solutions. For the detection of EWSR1 rearrangement, ZytoLight® EWSR1 Dual Color Break Apart Probe was used (Zytovision GmbH) following the manufacturer’s protocol. Slides were viewed under a fluorescent microscope equipped with multi-bandpass filters for the visualization of FISH foci.

**RT-qPCR**

RNA was extracted using the RNeasy mini kit (#74104, Qiagen), quantified using NanoDropTM 2000 spectrophotometer (ThermoFisher Scientific) and diluted to equal concentrations across all samples. Reverse-transcription was performed using a Transcriptor First Strand cDNA Synthesis Kit (#4896866001, Roche), following the manufacturers’ protocol.The cDNA was subjected to quantitative PCR using SYBR™ Select Master Mix (Thermo Fisher Scientific), and analyzed using the Applied Biosystems® ViiA7 real-time PCR system using the following program: denaturation at 95°C for 10 seconds, followed by annealing and then extension, both at 60°C for 30 seconds (40 cycles). The following gene-specific PCR primer sets were used (Integrated DNA Technologies): CCL5 (Hs.PT.58.1724551), CXCL10 (Hs.PT.58.3790956.g) and RPLP0 (Hs.PT.39a.22214824). For each sample, mRNA levels of target genes were normalized to the levels of RPLP0 mRNA, used as housekeeping control.

**Flow cytometry**

Cells were detached using Versene solution (Thermo Fisher Scientific), and pellets were washed with PBS. For PD-L1 cell-surface detection, primary antibody conjugation was performed by adding allophycocyanin (APC)-conjugated PD-L1 antibody or the corresponding isotype control followed by incubation for 20 min at 37ºC. Cells were stained with DAPI-containing PBS-FBS 2% (1 µg/mL DAPI for 15 min prior to analysis with an LSR II Flow Cytometer (BD Biosciences). DAPI was used as a viability marker and PD-L1-positive cells were gated on DAPI-negative cells only. For cell cycle analysis, cells were stained with Hoechst 3342 (1:1000 in PBS) for 20 min at 37ºC and then washed with PBS-FBS5% prior to fixation (4% PFA for 10 min at 4 ºC) and analysis with an LSR II Flow Cytometer (BD Biosciences).Data analysis was performed using the FlowJo software.

**JN1 xenografts pharmacological experiments**

All animal procedures were approved by the French “Ministère de l‘Enseignement supérieur, de la Recherche et de l’Innovation under CEEA 26 Project no. 2014-067-2328. A total of 32 mice were included in this study and were monitored for a total of 33 days following randomization. JN1 cells were subcutaneously implanted into the flank of 5-weeks-old NOD scid gamma (NSG) mice. When the tumor reached 40-50 mm3 threshold size, mice were randomly assigned to four treatment groups (n=8 per group), receiving either vehicle (control), talazoparib (0.2 mg/kg daily by oral gavage), M1774 (10mg/kg 2qw by oral gavage), or a combination of both. Tumor size was monitored twice weekly during four weeks using calipers.

**Supplementary Figures**

**Supplementary Figure S1**

**
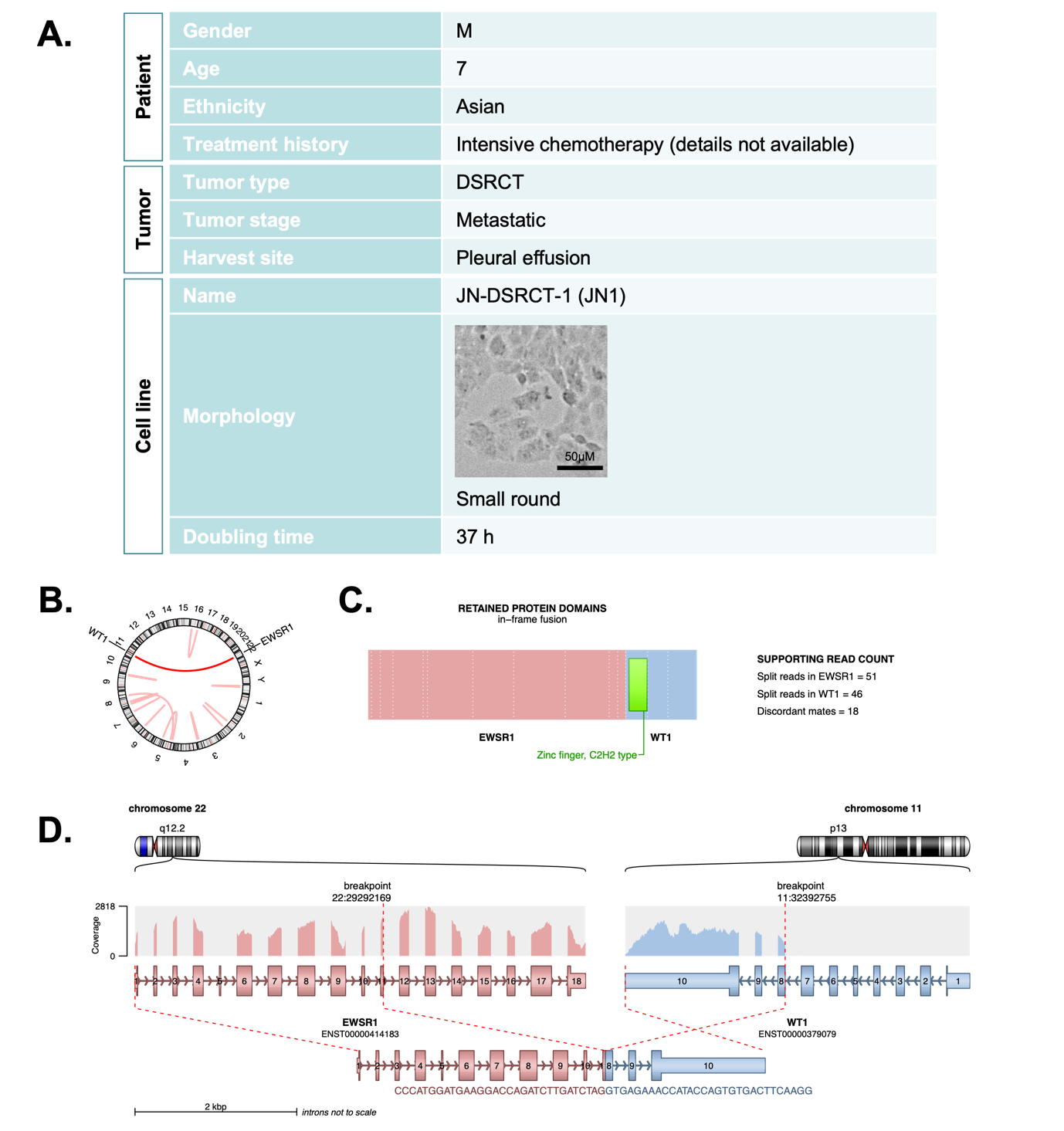
**

**Supplementary Figure S1. JN1 cell line characteristics. A.** Origin and characteristics of the patient-derived JN1 cell line. **B-D.** Molecular characterization and visualization of the EWS-WT1 fusion found in the JN1 cell line by circos plot (B), protein view (C) and chromosome view (D).

**Supplementary Figure S2**

**
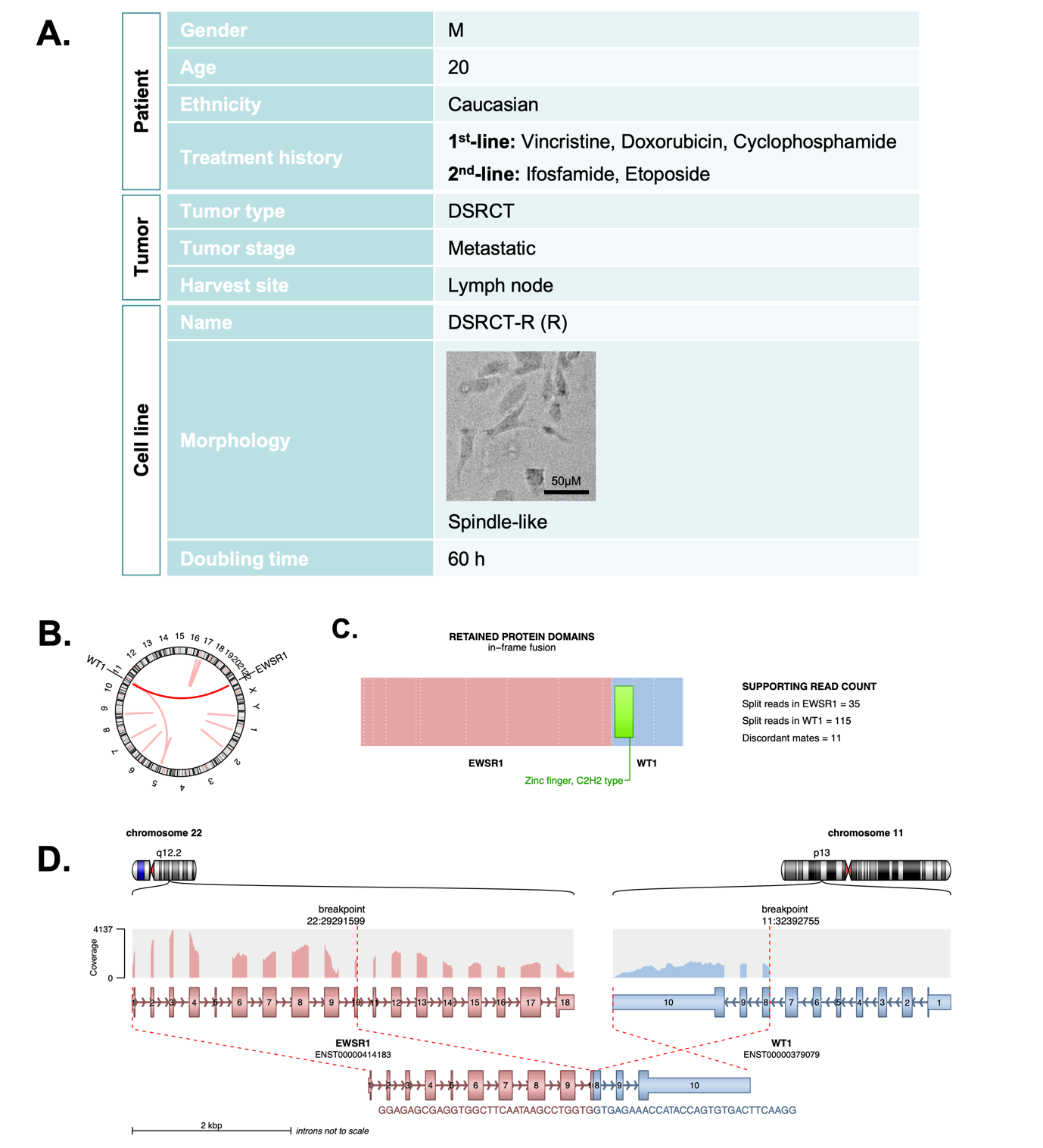
**

**Supplementary Figure S2. R cell line characteristics. A.** Origin and characteristics of the patient-derived R cell line. **B-D.** Molecular characterization and visualization of the EWS-WT1 fusion found in the R cell line by circos plot (B), protein view (C) and chromosome view (D).

**Supplementary Figure S3**

**
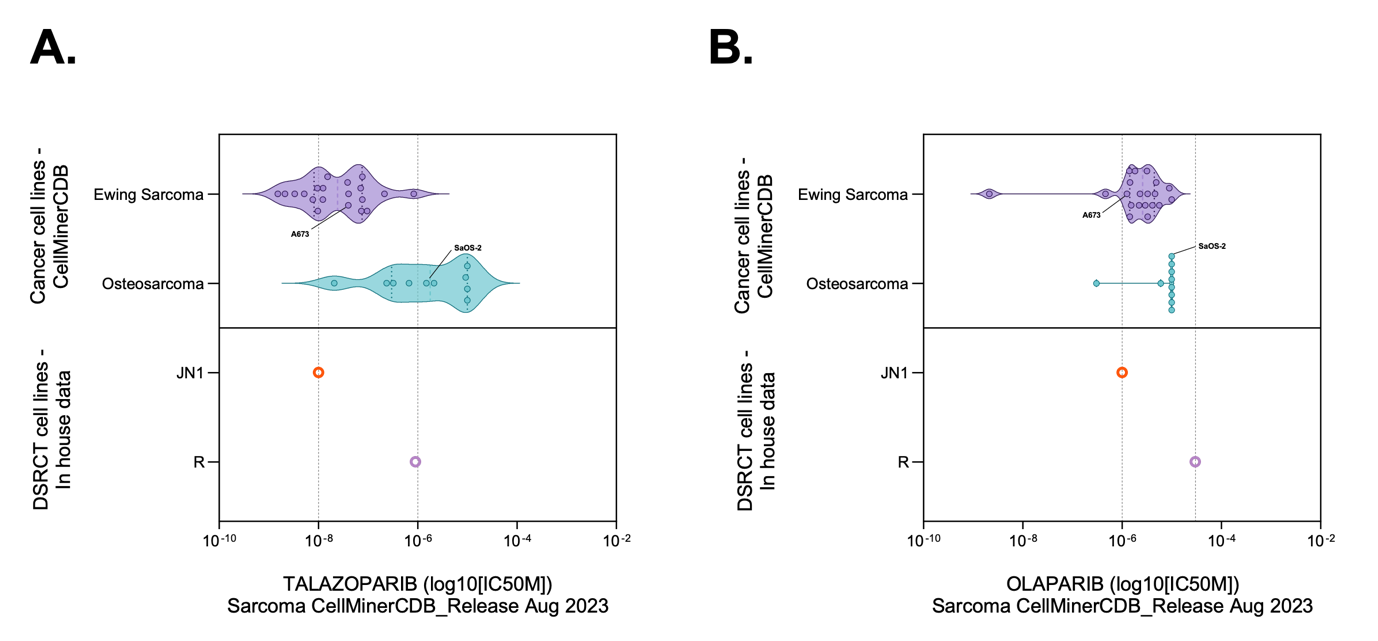
**

**Supplementary Figure S3, related to Figure 1. Sensitivity to PARP inhibitors of the JN1 and R cell lines as compared with sarcoma cell lines in the SarcomaMiner project database. A, B.** Violin plots showing the relative sensitivity (log_10_ of SF50) of Ewing sarcoma and osteosarcoma cell lines exposed to the PARP inhibitor talazoparib (A) or olaparib (B) at concentrations ranging from 1.5 nM to 10 µM for 4 days in the SarcomaMiner database, in comparison with that of the JN1 and R DSRCT cell lines (highlighted with dashed lines). JN1 and R cell lines sensitivities were extrapolated from the survival assays presented in Fig. 1C and Fig. 1D; the corresponding SF50 were determined using a four-parameter logistic dose-response curve followed by log_10_-transformation, to ensure results comparability. Ewing sarcoma cell lines (n=20): A673, CHLA-10, CHLA-25, CHLA-258, CHLA-32, CHLA-9, COG-E-352, ES1, ES2, ES3, ES4, ES6, ES7, ES8, EW8, RD-ES, SK-ES-1, SK-N-MC, TC-32, TC-71; Osteosarcoma cell lines (n=12): CHA-59, HOS, Hs870.T, Hu09, KHOS-NP, KHOS-240S, KHOS-312H, OHS, SaOS-2, SJSA-1, T1-73, U-2OS.

**Supplementary Figure S4**

**
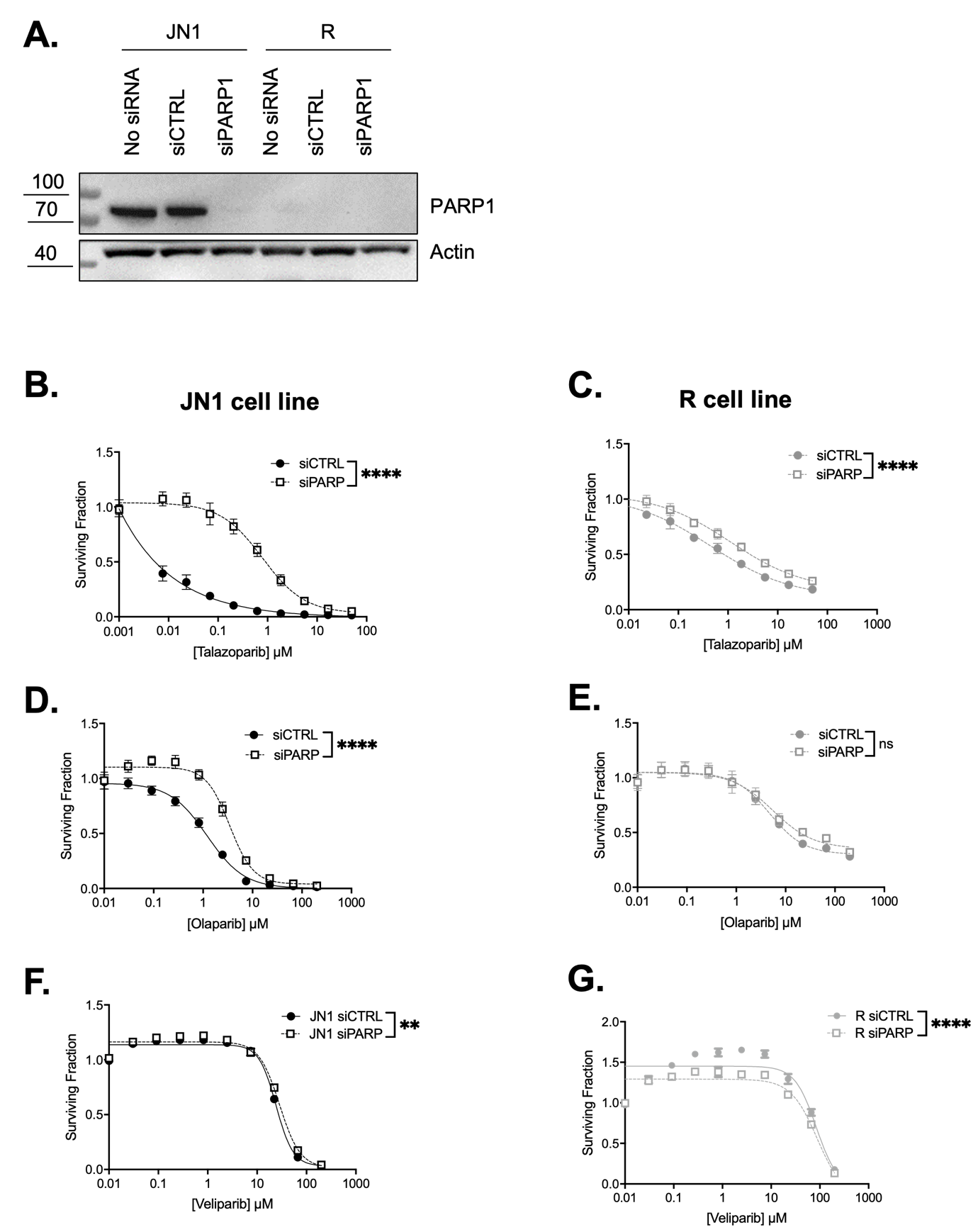
**

**Supplementary Figure S4, related to Figure 1. Role of PARP1 in the sensitivity of DSRCT cells to PARP inhibitors. A.** Western blot of PARP1 in JN1 and R cells exposed to siRNA-mediated silencing of PARP1. Cells were transfected with either siCNTRL or siPARP1, and whole-cell lysates were prepared 48 h post-transfection. **B-G**. Dose-response survival curves of PARP inhibitors in JN1 (B, D, F) and R (C, E, G) cells exposed to siRNA-mediated silencing of PARP1. Cells were transfected with either siCNTRL or siPARP1, and exposed 48 h post-transfection to increasing concentrations of talazoparib (B, C), olaparib (D, E), or veliparib (F, G) for 7 days in short term survival assay, after which cell survival was assessed by use of CellTiter Glo®. Mean ± SD; *n* = 3; two-way ANOVA and *post hoc* Šídák’s test.

**Supplementary Figure S5**

**
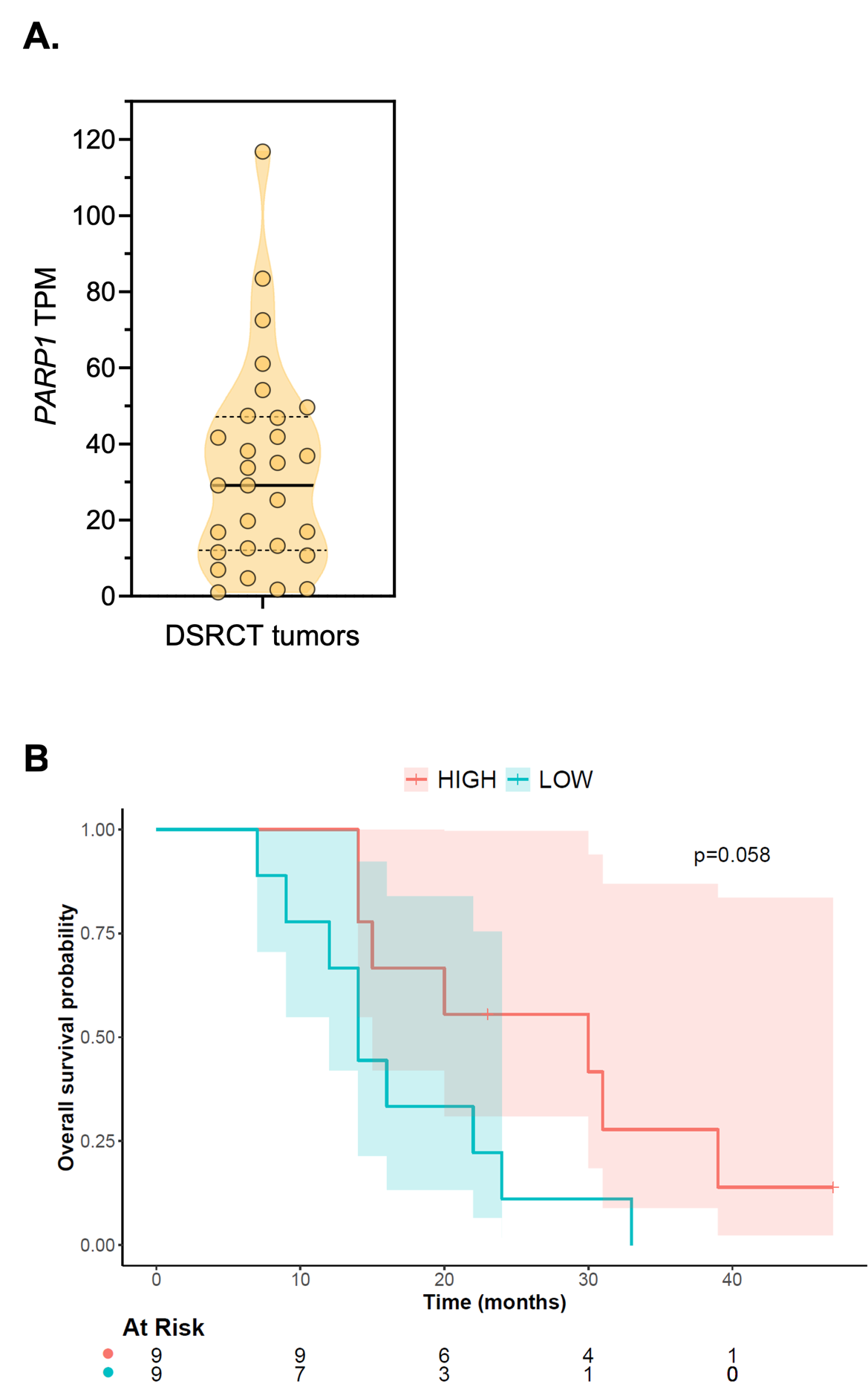
**

**Supplementary Figure S5, related to Figure 2. PARP1 expression in DSRCT patients’ tumor samples and its correlation with overall survival. A.** *PARP1* expression as assessed by RNA-seq in a cohort of 29 tumor samples from patients with DSRCT; TPM, transcripts per million. **B.** Kaplan-Meier survival curves of patients with DSRCT according to *PARP1* expression in their tumor (n=29; based on A; *PARP1*-low, lower quartile of expression; *PARP1*-high, upper quartile of expression); log-rank test p=0.058.

**Supplementary Figure S6**


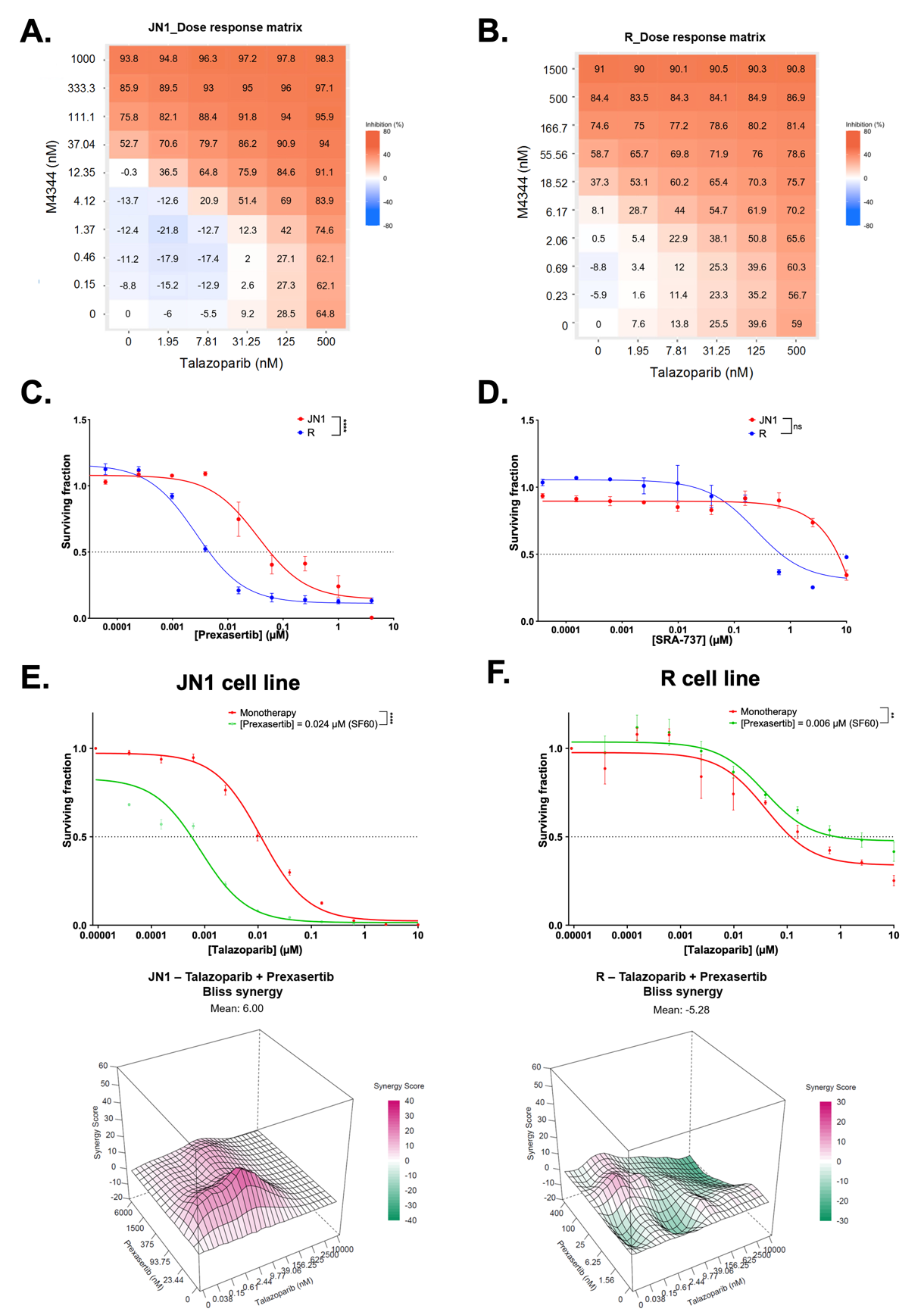


**Supplementary Figure S6, related to Figure 2. Synergy analysis of the PARP plus CHK1 inhibitor combination in JN1 and R cell lines. A, B.** Dose-response matrix of the talazoparib – M4344 combination in JN1 (A) and R (B) cell lines, related to the surface plots presented in Fig. 2C, D. The x- and y-axes values indicate drug concentrations for talazoparib and M4344 respectively; matrix values indicate the score of inhibition corresponding to each combination. **C, D**. Dose-response survival curves of the JN1 and R cell lines exposed to increasing concentrations of the CHK1 inhibitors prexasertib (C) or SRA-737 (D) for 7 days in short-term survival assay. Mean ± SD; *n* = 3; two-way ANOVA. **E, F**. Dose-response survival curves and surface plots of Bliss synergy scores calculated for the talazoparib – prexasertib combination in JN1 (E) and R (F) cell lines. Cells were exposed to increasing concentrations of talazoparib and prexasertib for 7 days in short-term survival assay. The x- and y-axes values indicate drug concentrations, and the z-axis values the associated synergy score. Score < -10, antagonistic interaction; score = 0, absence of interaction; score > 10, synergistic interaction. Mean ± SD; *n* = 3; two-way ANOVA.

**Supplementary Figure S7**

**
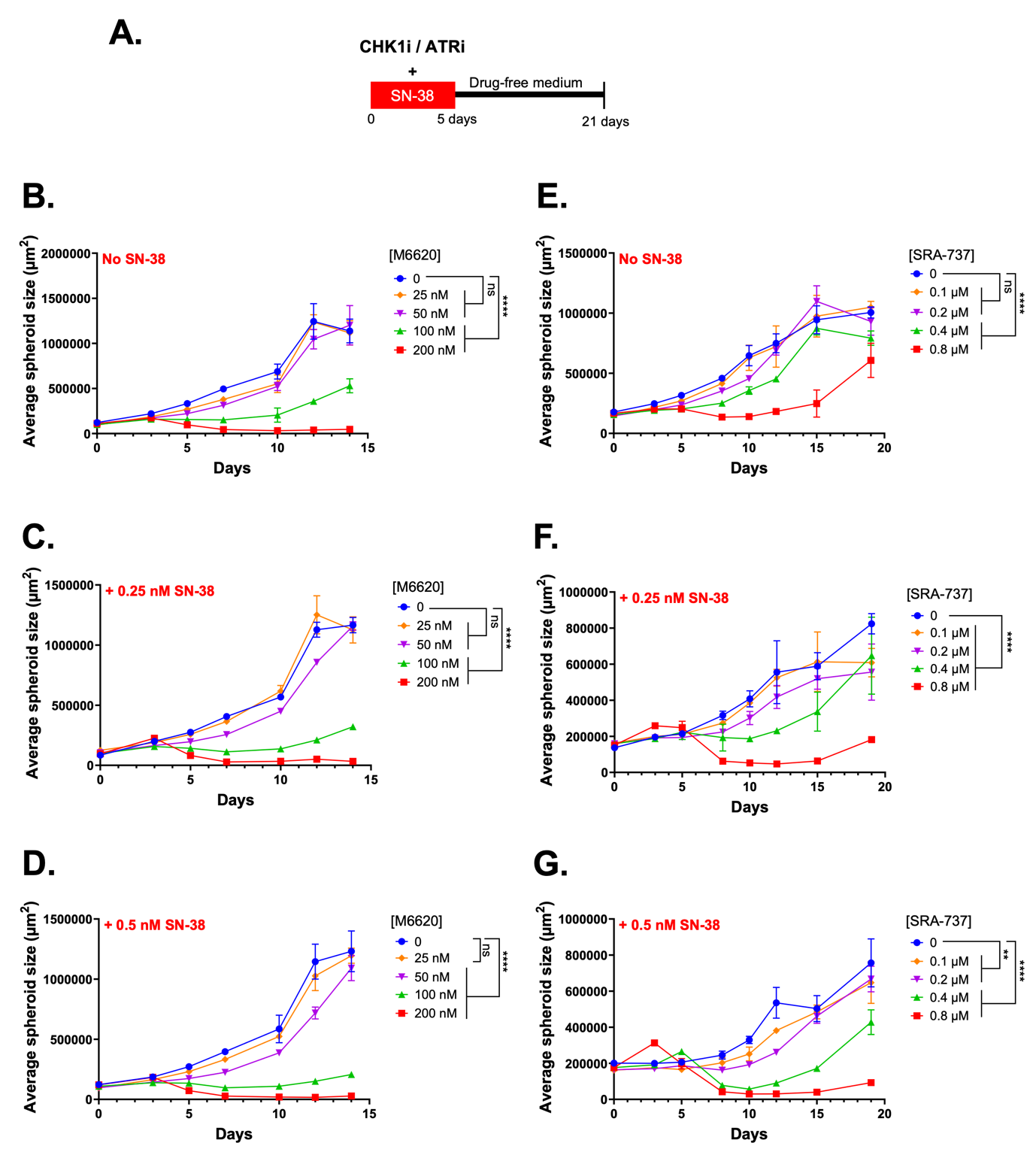
**

**Supplementary Figure S7, related to Figure 2. Combination of SN-38 with the ATRi M6620 and CHK1i SRA-737 in JN1 spheroids. A.** Schematics describing the experiment layout. **B-E.** Kinetics of JN1 spheroid growth following exposure to ATRi or CHK1i in the presence or absence of SN-38. Spheroids were exposed to increasing concentrations of the ATRi M6620 (B-D) or CHK1i SRA-737 (E-G) for 5 days with or without SN-38 (upper panel, no SN-38; middle panel, 0.25 nM SN-38; bottom panel, 0.5 nM SN-38), after which the drugs were removed and spheroids size was monitored for up to 19 days post-treatment initiation. Mean ± SD; *n* = 6 two-way ANOVA and *post hoc* Šídák’s test.

**Supplementary Figure S8
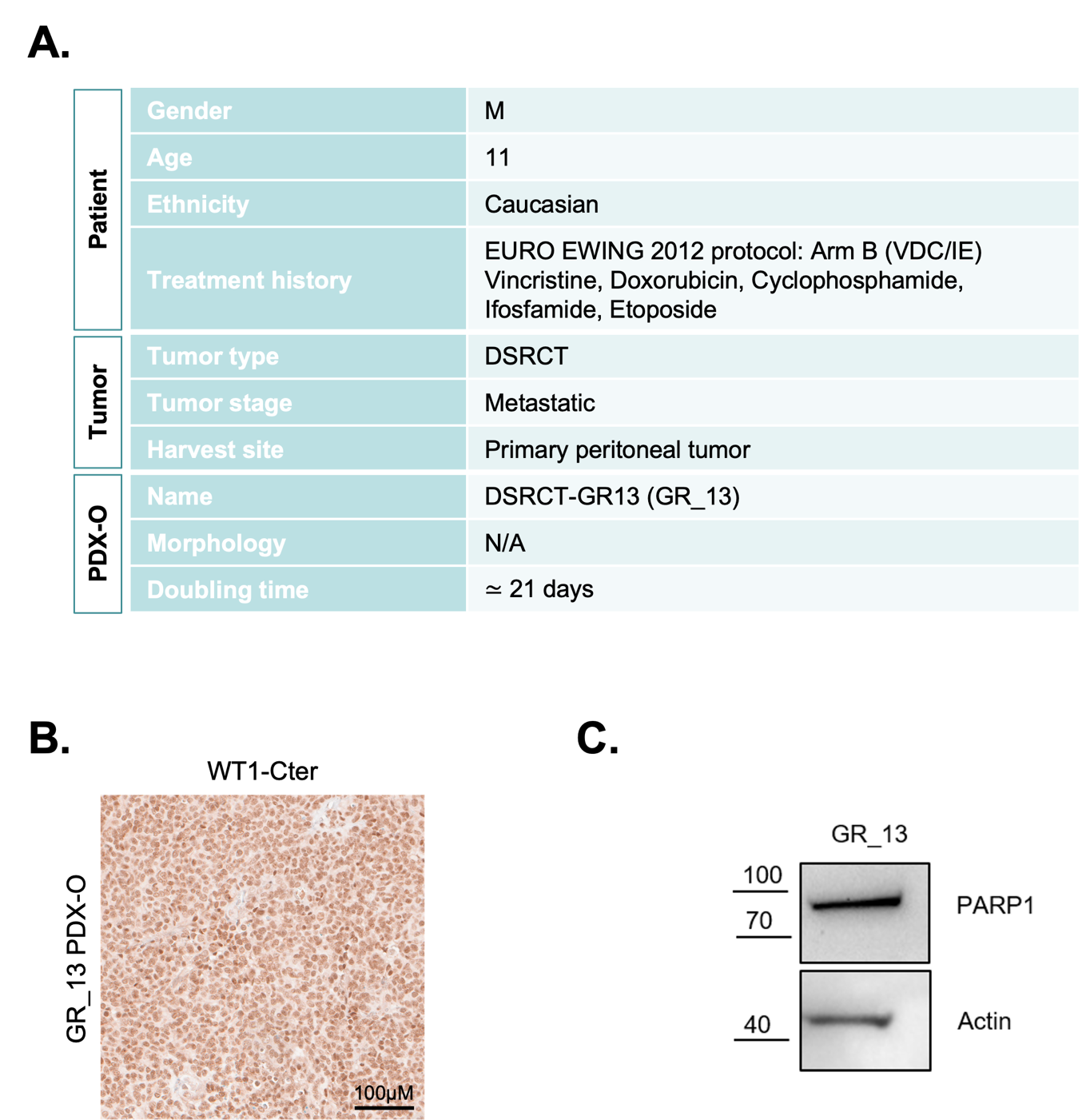
Supplementary Figure S8, related to Figure 2. GR_13 PDX-O model characteristics. A.** Origin and characteristics of the patient-derived xenograft (PDX)-derived organoid GR_13. **B.** Validation of EWS-WT1 protein expression in the GR_13 PDX-O by WT1-Cter immunohistochemistry. Since wildtype WT1 is reportedly not expressed in DSRCT (Hénon et al, *Cell Rep Med* 2024), WT1-Cter was used as a marker to confirm the presence of EWS-WT1 protein expression. **C.** Western blot of PARP1 in the GR_13 model as compared with the JN1 and R cell lines.

**Supplementary Figure S9**

**
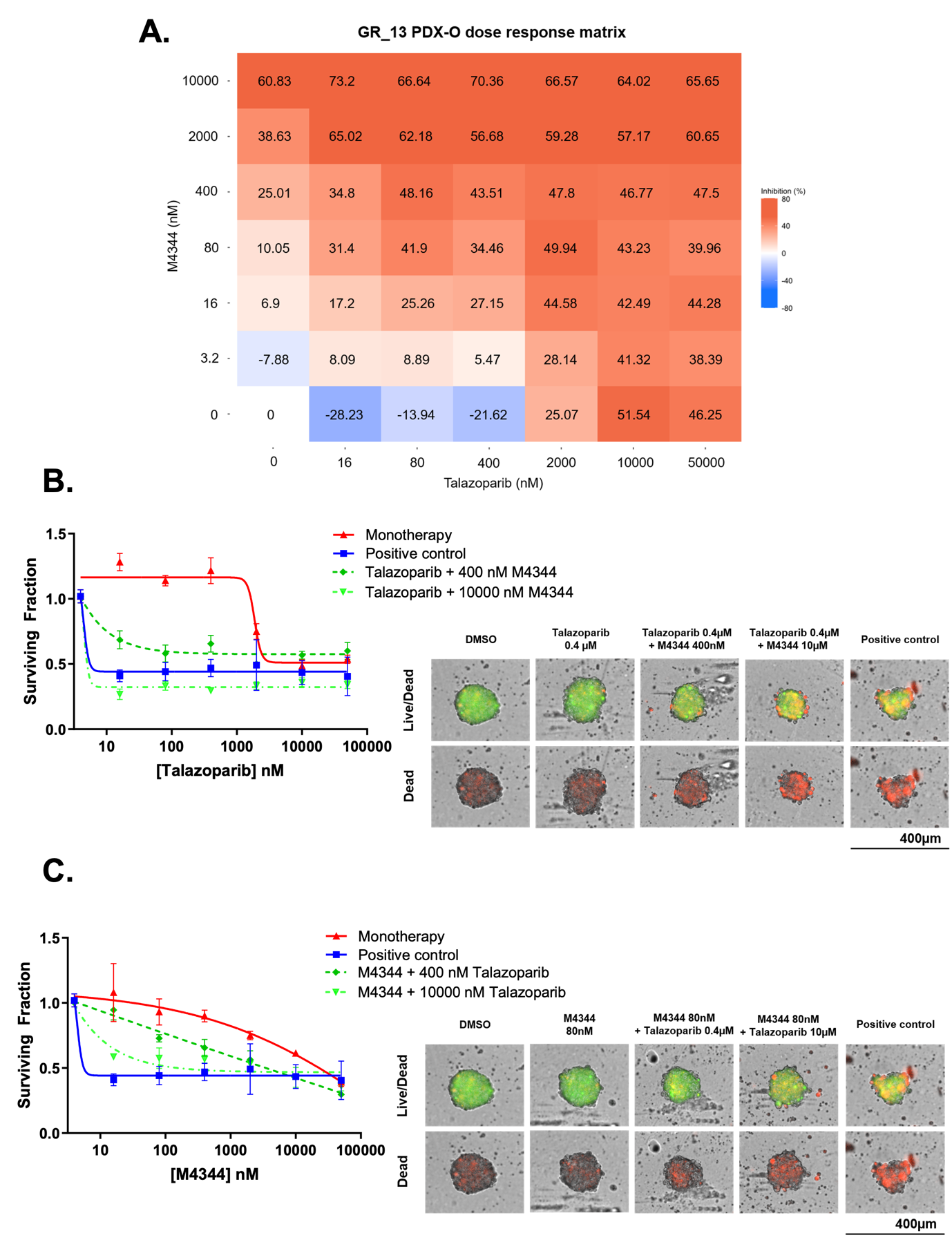
Supplementary Figure S9, related to Figure 2. Synergy analysis of the PARP plus ATR inhibitor combination in GR_13 PDX-O. A**. Dose-response matrix of the talazoparib – M4344 combination in the GR_13 PDX-O, related to the surface plots presented in Fig. 2F. The x- and y-axes values indicate drug concentrations for talazoparib and M4344 respectively; matrix values indicate the score of inhibition corresponding to each combination. **B, C.** Dose-response survival curves of the talazoparib – M4344 combination with talazoparib dose-response and M4344 fixed dose (B) or M4344 dose-response and talazoparib fixed dose (C). Representative images of organoids stained with the Cyto3D Live-Dead assay are shown to the right. Green, live cells; red, dead cells. Positive control: topotecan, 1 µM.

**Supplementary Figure S10
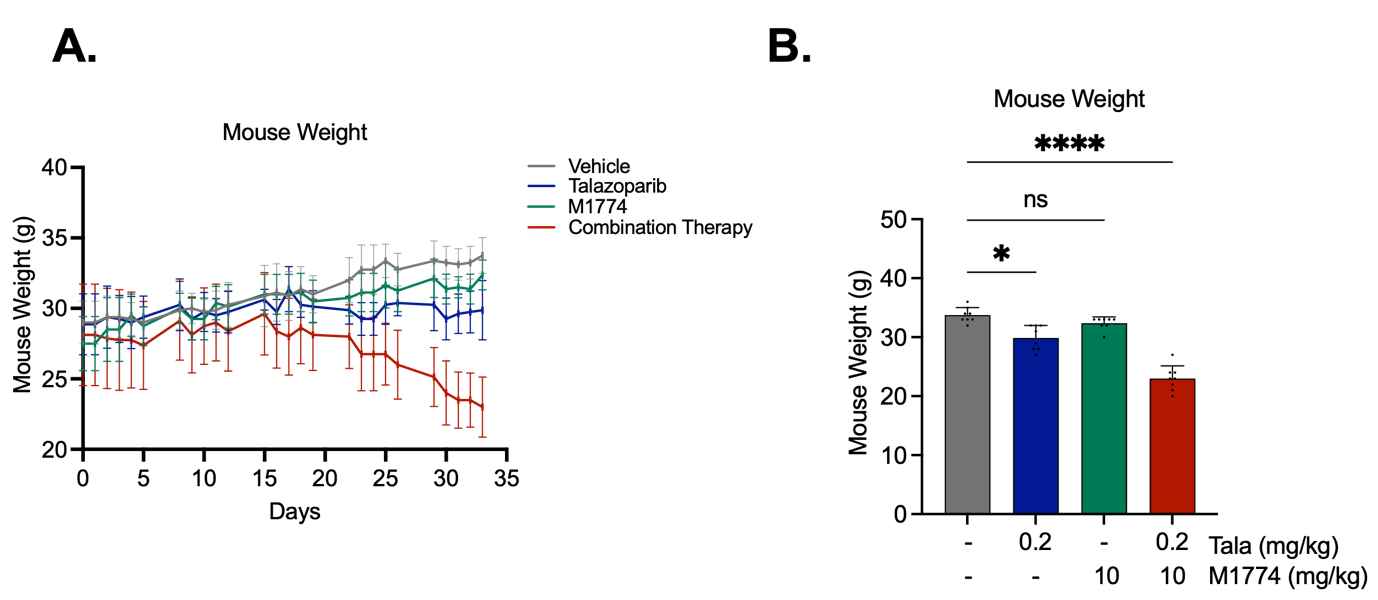
**

**Supplementary Figure S10, related to Figure 2. *In vivo* evaluation of the PARP plus ATR inhibitor combination in JN1 xenografts. A**. Evolution of JN1 xenograft-bearing mice body weight over time. Mean tumor volume ± SD; two-way ANOVA and post hoc Dunnett's test. **B.** Body weight of mice at the time of sacrifice day. Mean ± SD; one-way analysis of variance and *post hoc* Šídák’s test.

**Supplementary Figure S11**


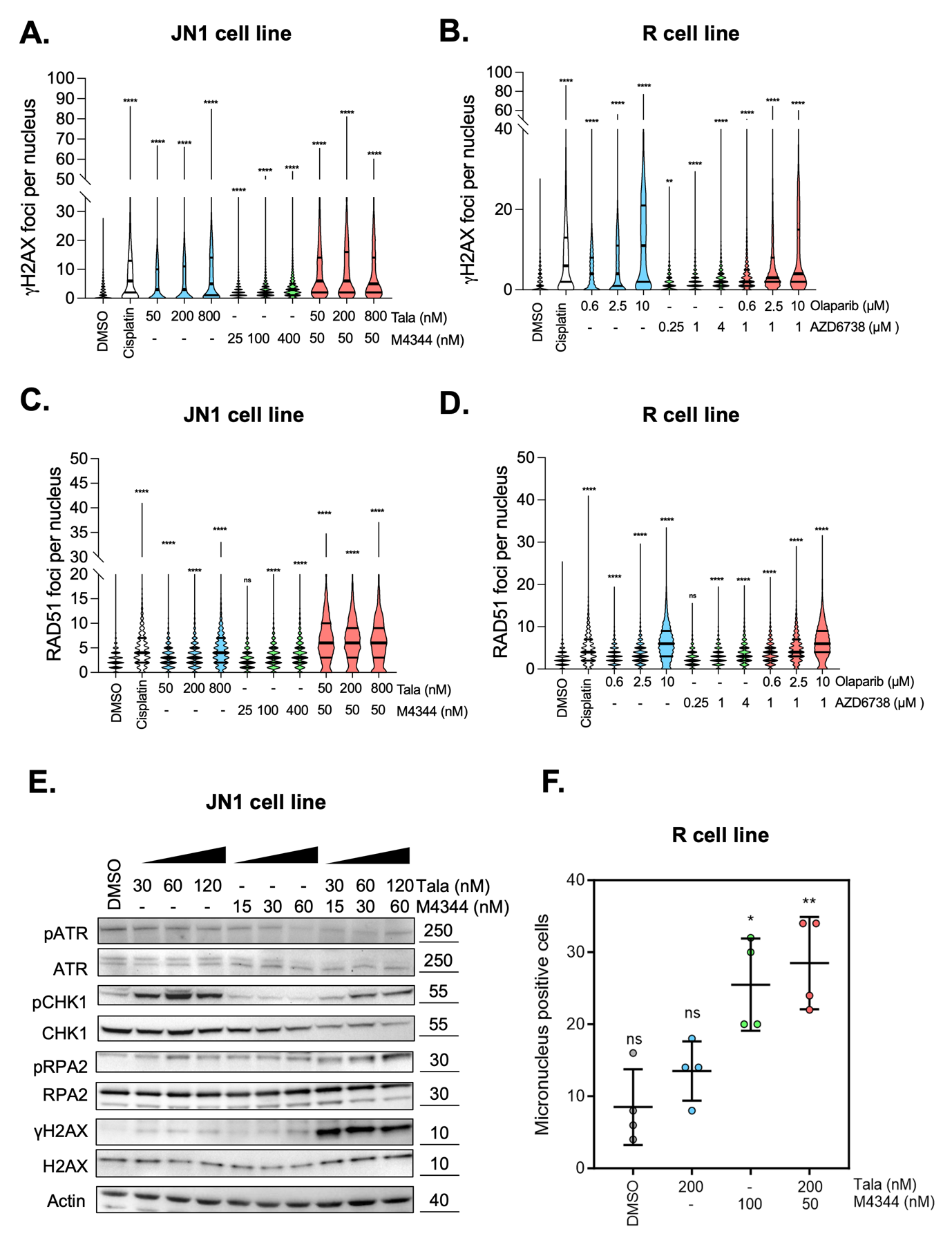


**Supplementary Figure S11, related to Figure 3. DNA damage response analysis of DSRCT cells exposed to PARP and ATR inhibitors. A-D.** Automated quantification of γH2AX (A, C) or RAD51 (C, D) in JN1 cells exposed to PARP inhibitor, ATR inhibitor or a combination of both. Cells were and exposed to DMSO control, PARP inhibitor talazoparib, ATR inhibitor M4344, or a combination of both for 72 h. A minimum of 500 nuclei were analyzed for each condition. Data from one representative experiment of three biological replicates is shown. Violin plots show the absolute number of foci per nucleus. The thick line indicates median; the thin lines indicate lower and upper quartiles; two-way ANOVA and *post hoc* Dunn’s test. The data presented are an extension of Fig. 3A. **E.** Western blot of pATR, ATR, pCHK1, CHK1, pRPA2, RPA2, γH2AX and H2AX in JN1 cells upon PARP inhibitor, ATR inhibitor or a combination of both. Whole-cell lysates were generated from cells exposed for 48 h to DMSO (vehicle control), a concentration range of talazoparib, M4344 or a combination of both. **F.** Quantification of micronuclei-positive cells in PicoGreen®-stained R cells upon PARP inhibitor, ATR inhibitor or a combination of both. Cells were exposed for 6 days to either or both compounds. Scatter plot showing the percentage of micronuclei-positive cells. Four independent fields cells were analyzed with 50 cells quantified per field (n=200). Median ± SD; n = 4; one-way ANOVA and post hoc Dunn’s test.

**Supplementary Figure S12**
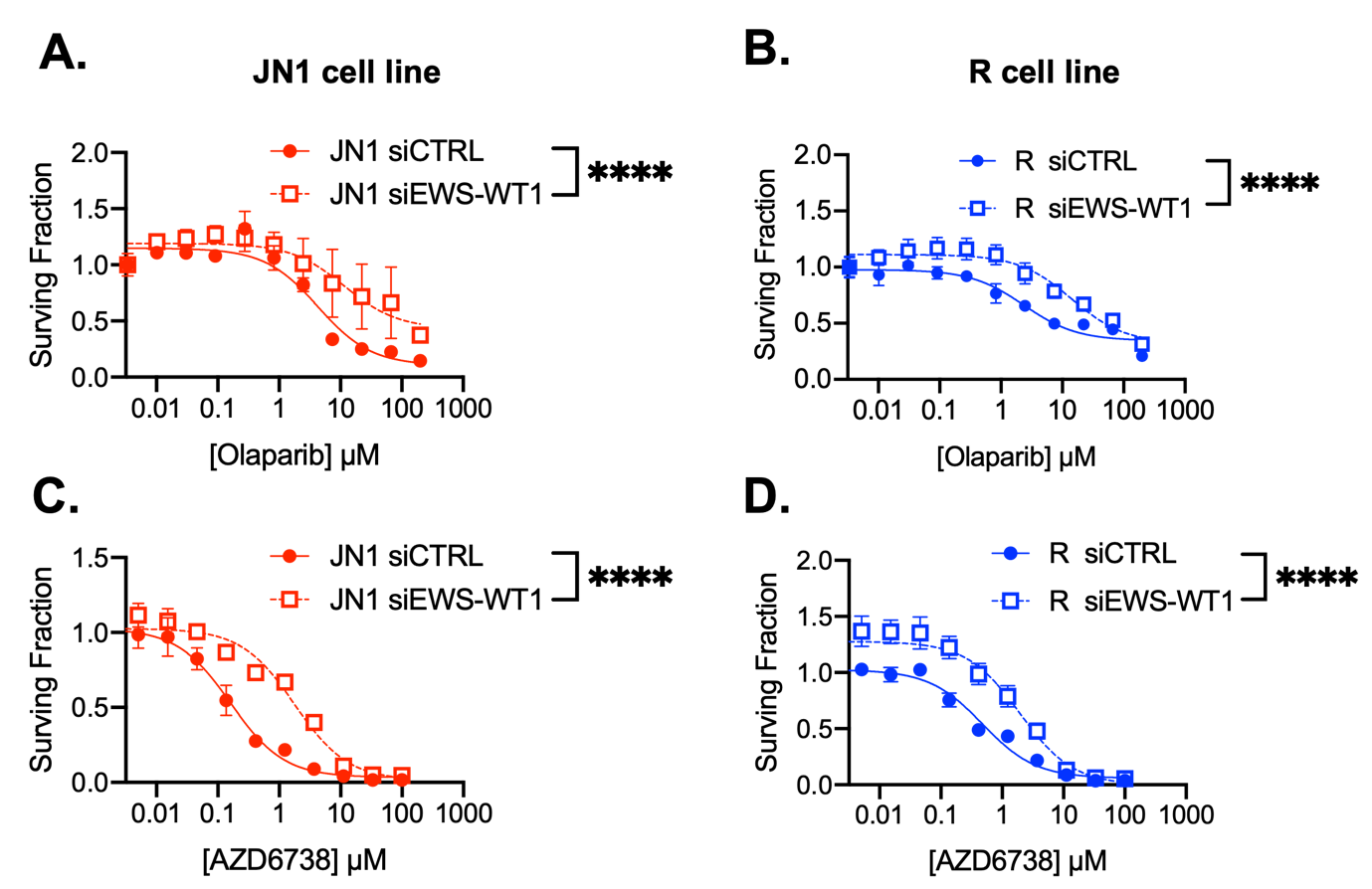


**Supplementary Figure S12, related to Figure 4. Role of EWS-WT1 in the sensitivity to PARP and ATR inhibitors. A-D.** Dose-response survival curves of JN1 (A, C) or R (B, D) cells exposed to PARP or ATR inhibitor in the presence or absence of siRNA-mediated silencing of EWS-WT1. Cells were transfected with siCNTRL or siEWS-WT1 and exposed, 48h post-transfection, to increasing concentrations of olaparib (A, B) and AZD6738 (C, D) for 7 days in short-term survival assay, after which cell survival was assessed by CellTiter Glo®. Mean ± SD; *n* = 3; two-way ANOVA and *post hoc* Šídák’s test.

**Supplementary Figure S13**


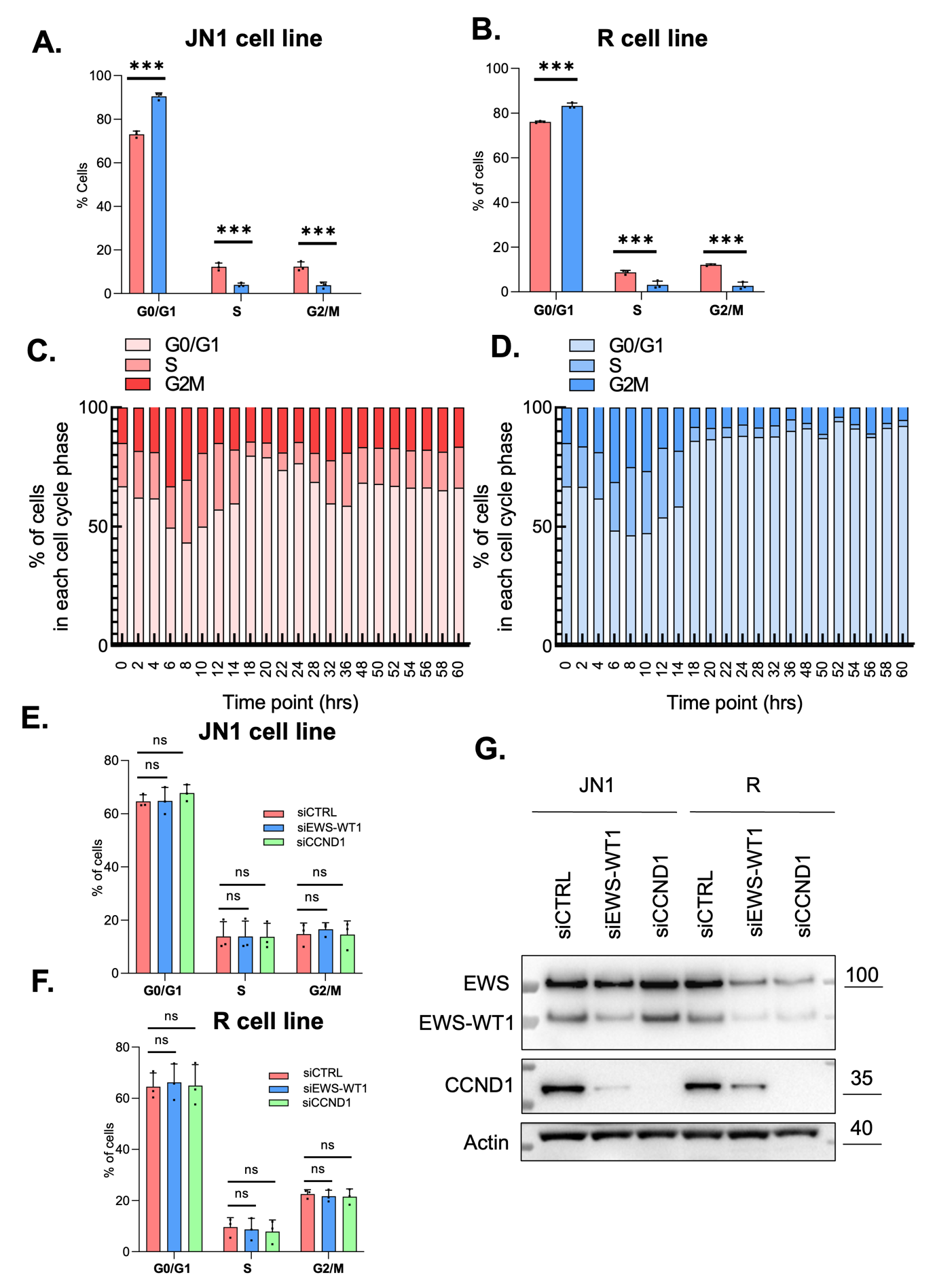


**Supplementary Figure S13, related to Figure 4. Cell cycle analysis of DSRCT cells in presence or absence of EWS-WT1 silencing. A, B.** Bar plots displaying the percentage of JN1 (A) or R (B) cells in each cell cycle phase, following transfection with siCNTRL (red bars) of siEWS-WT1 (blue bars), 48 h post-transfection. Mean ± SD, *n*=3; Paired Student’s t-test, two-tailed. **C, D.** Bar plots displaying a kinetics of the percentage of JN1 cells in each cell cycle phase following transfection with siCNTRL (C) of siEWS-WT1 (D). Mean ± SD, *n*=3. **E, F.** Bar plots displaying the percentage of JN1 (E) or R (F) cells in each cell cycle phase, following transfection with siCNTRL (red bars), siEWS-WT1 (blue bars) or siCCND1 (green bars), 14 h post-transfection. Mean ± SD, *n*=3; two-way ANOVA. (**G**) Western blot of JN1 and R cells exposed to siRNA-mediated silencing of EWS-WT1 or CCND1. Cells were transfected with either siCNTRL, siEWS-WT1 or siCCND1, and whole-cell lysates were prepared 14 h post-transfection.

**Supplementary Figure S14**


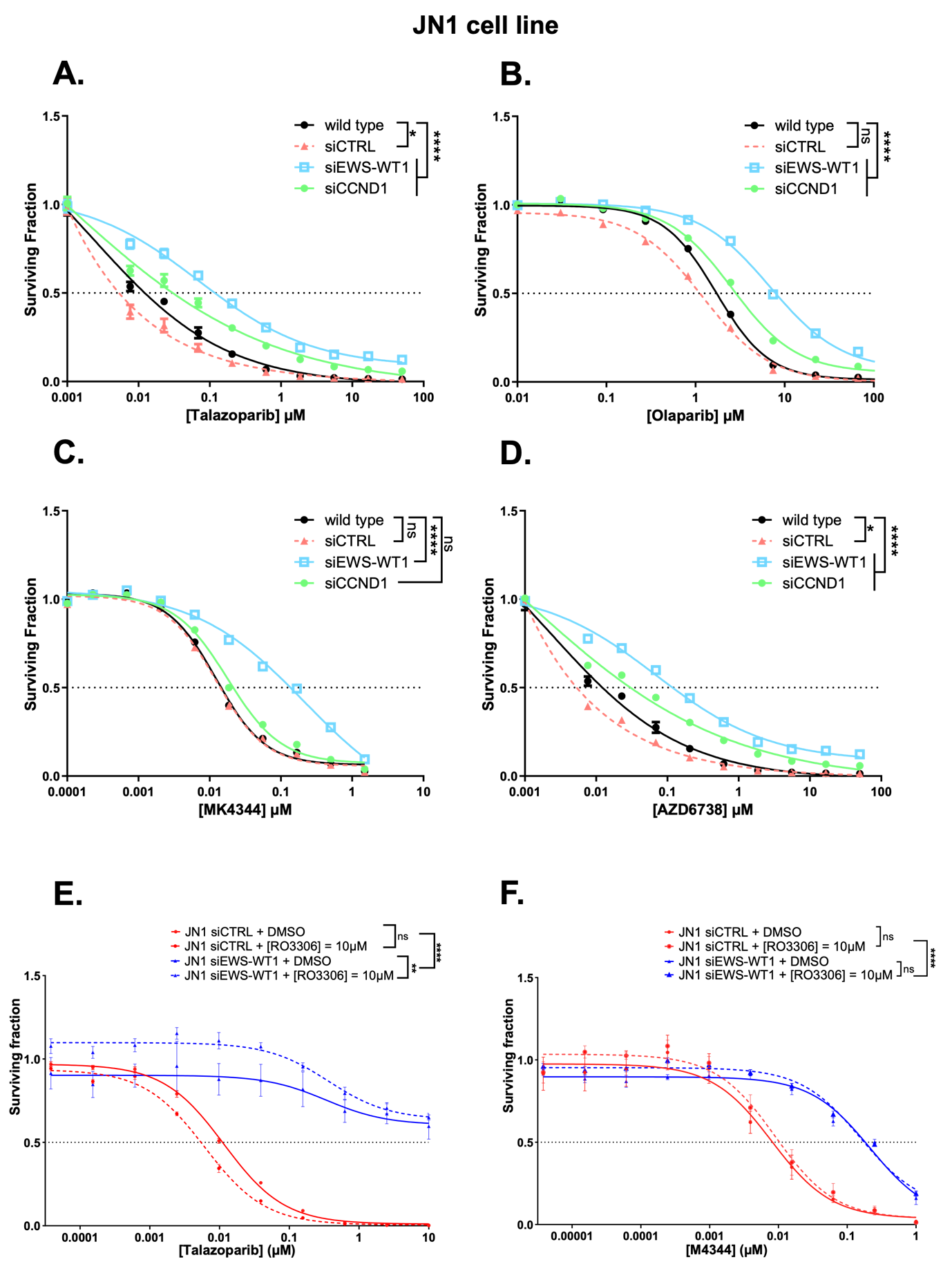


**Supplementary Figure S14, related to Figure 4. Effect of cell cycle arrest on the sensitivity of JN1 cells to PARP and ATR inhibitors. A-D.** Dose-response survival curves of JN1 cells exposed to PARP or ATR inhibitors in the presence or absence of siRNA-mediated silencing of EWS-WT1 or CCND1. Cells were transfected with siCNTRL, siEWS-WT1 or siCCND1 and exposed, 48h post-transfection, to increasing concentrations of talazoparib (A), olaparib (B), M4344 (C) or AZD6738 (D) for 7 days in short-term survival assay, after which cell survival was assessed by CellTiter Glo®. Mean ± SD; *n* = 3; two-way ANOVA and *post hoc* Šídák’s test. **E, F**. Dose-response survival curves of JN1 cells exposed to ATR or PARP inhibitors in the presence or absence of CDK1 inhibition and siRNA-mediated silencing of EWS-WT1. Cells were transfected with siCNTRL or siEWS-WT1, and exposed to increasing concentrations of the PARP inhibitor Talazoparib (E) or ATR inhibitor M4344 (F) for 7 days with addition of the CDK1 inhibitor RO-3306 for 72 h, after which cell survival was assessed by CellTiter Glo®. Mean ± SD; *n* = 3; two-way ANOVA and *post hoc* Šídák’s test.

**Supplementary Figure S15**


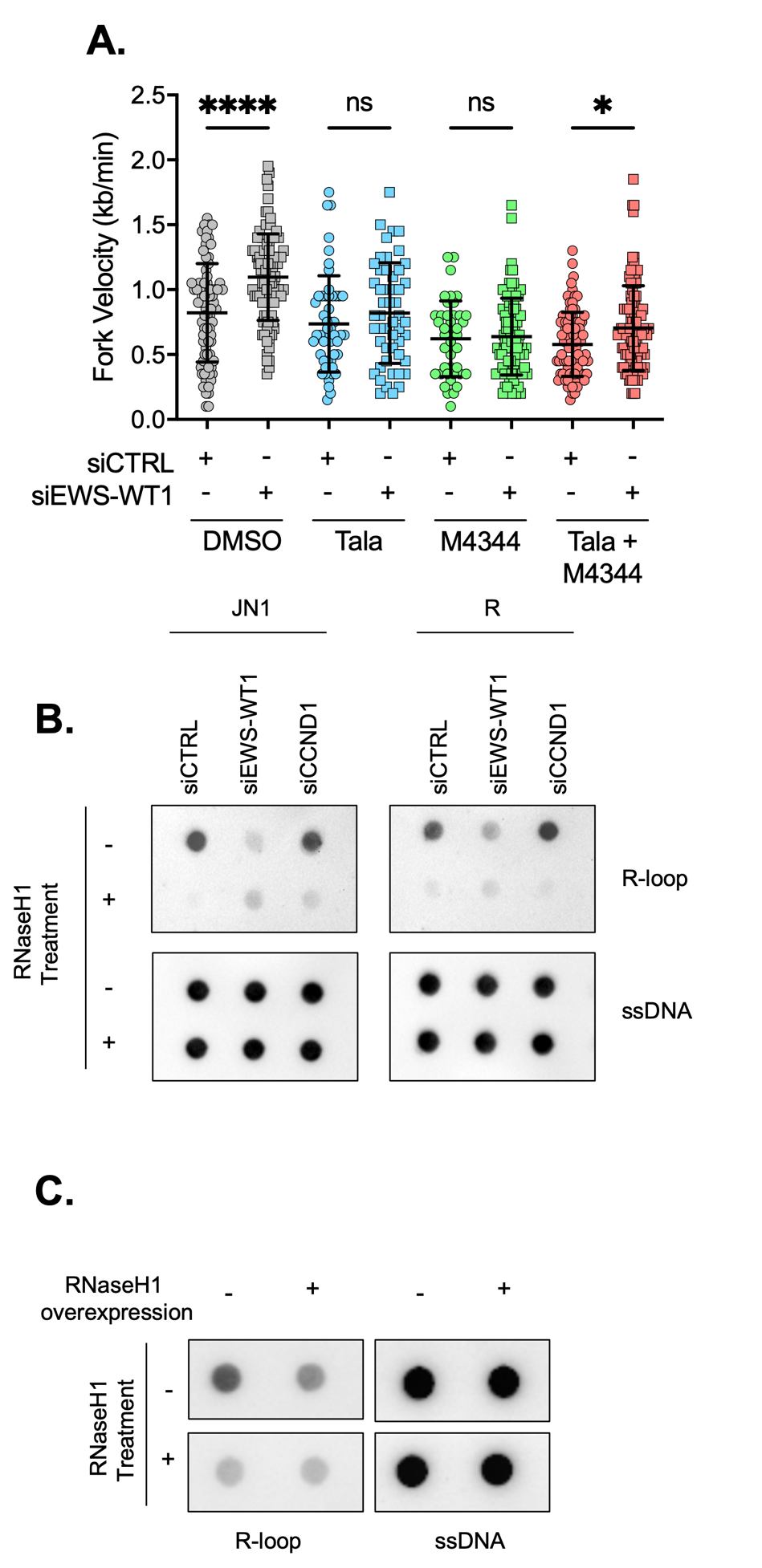


**Supplementary Figure S15, related to Figure 5. Effect of EWS-WT1 silencing on replication fork progression and R-loop formation in DSRCT cells. A.** Assessment of replication fork speed (kb/min) in JN1 cells upon PARP inhibitor, ATR inhibitor, or a combination of both in the presence or absence of siRNA-mediated silencing of EWS-WT1. Synchronized cells were transfected with siCNTRL or siEWS-WT1 and exposed, 8 h post-transfection, to DMSO control, PARP inhibitor talazoparib, ATR inhibitor M4344 or a combination of both for 6 h. A minimum of 50 forks per condition were analyzed. Mean ± SD, each dot represents a single replication fork; *n* = 2, two-way ANOVA and *post hoc* Šídák’s test. The data presented are an extension of Fig. 5B. **B.** DNA:RNA hybrid dot-blot of genomic DNA extracted from JN1 (left) or R cells (right) subjected to siRNA-mediated silencing of EWS-WT1 or CCND1. Synchronized cells were transfected with siCNTRL, siEWS-WT1 or siCCND1 and genomic DNA was extracted 14 h post-transfection. S9.6 antibody was used to detect RNA:DNA hybrids and ssDNA antibody was used as a loading control. **C.** DNA:RNA hybrid dot-blot of genomic DNA extracted from JN1 cells in the presence or absence of RNaseH1 overexpression. Genomic DNA was extracted from JN1 and RNaseH1-overexpressing JN1 cells. S9.6 antibody was used to detect RNA:DNA hybrids and ssDNA antibody was used as a loading control.

**Supplementary Figure S16
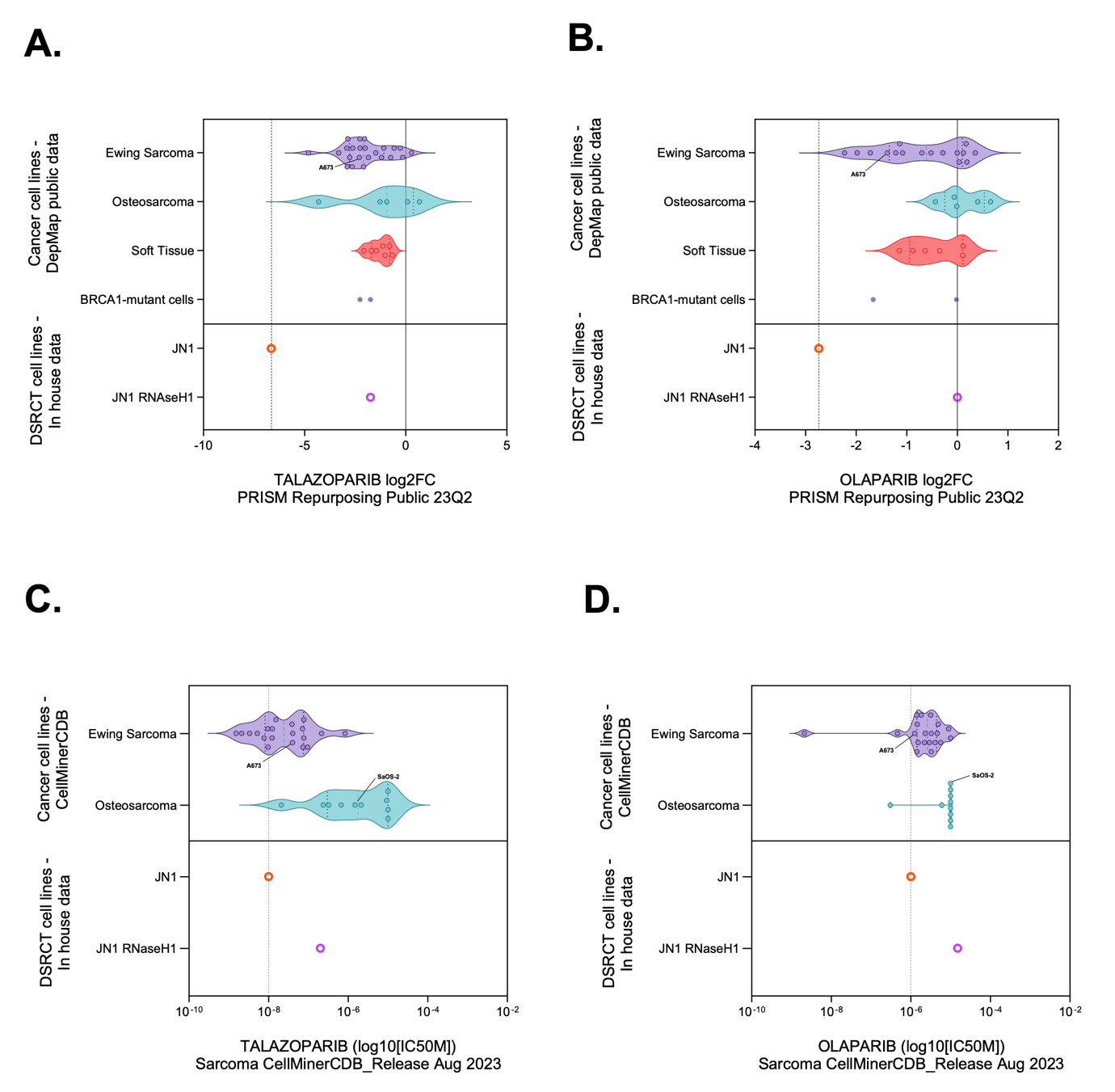
Supplementary Figure S16, related to figure 5. Sensitivity to PARP inhibitors of the JN1 and JN1-RNaseH1 cell lines as compared with sarcoma cell lines in the DepMap and SarcomaMiner project databases.** **A, B.** Violin plots showing the relative sensitivity (log_2_ fold-change of cell viability) of cell lines exposed to the PARP inhibitor talazoparib (A) or olaparib (B) (Prism Repurposing 23Q2) after a single-dose exposure at 2.5µM for 5 days in the DepMap database. JN1 and JN1-RNaseH1 cell lines sensitivities were extrapolated from the survival assays presented in Fig. 5F and Fig. 5G; surviving fractions were calculated at 2.5µM and log_2_ transformed. Ewing Sarcoma cell lines (n=23): RDES, A673, SKES1, CADOES1, EWS502, MHHES1, EW8, A673STAG2KO16, A673STAG2KO45, A673STAG2NT14, A673STAG2NT23, CBAGPN, CHLA10, SKNEP1, SKPNDW, TC32; Osteosarcoma cell lines (n=5): G292CLONEA141B1, MG63, U2OS, HOS, SJSA1; Soft-tissue sarcoma cell lines (n=7): S117, TE617T, HT1080, HS729, RD, RKN, RH30, including rhabdomyosarcoma (n=4), leiomyosarcoma (n=1), fibrosarcoma (n=1) and NOS sarcoma cell lines (n=1). The *BRCA1/2*-mutant IGROV1 ovarian cancer cell line and *BRCA1*-mutant MDA-MB-436 breast cancer cell line were used as positive controls for sensitivity to PARP inhibitors. **C, D.** Violin plots showing the relative sensitivity (log_10_ of SF50) of Ewing sarcoma and osteosarcoma cell lines exposed to the PARP inhibitor talazoparib (C) or olaparib (D) at concentrations ranging from 1.5 nM to 10 µM for 4 days in the SarcomaMiner database, in comparison with that of the JN1 and JN1-RNaseH1 cell lines. JN1 and JN1-RNaseH1 cell lines sensitivities were extrapolated from the survival assays presented in Fig. 5F and Fig. 5G; the corresponding SF50 were determined using a four-parameter logistic dose-response curve followed by log_10_-transformation, to ensure results comparability. Ewing sarcoma cell lines (n=20): A673, CHLA-10, CHLA-25, CHLA-258, CHLA-32, CHLA-9, COG-E-352, ES1, ES2, ES3, ES4, ES6, ES7, ES8, EW8, RD-ES, SK-ES-1, SK-N-MC, TC-32, TC-71; Osteosarcoma cell lines (n=12): CHA-59, HOS, Hs870.T, Hu09, KHOS NP, KHOS-240S, KHOS-312H, OHS, SaOS-2, SJSA-1, T1-73, U-2OS.

**Supplementary Figure S17
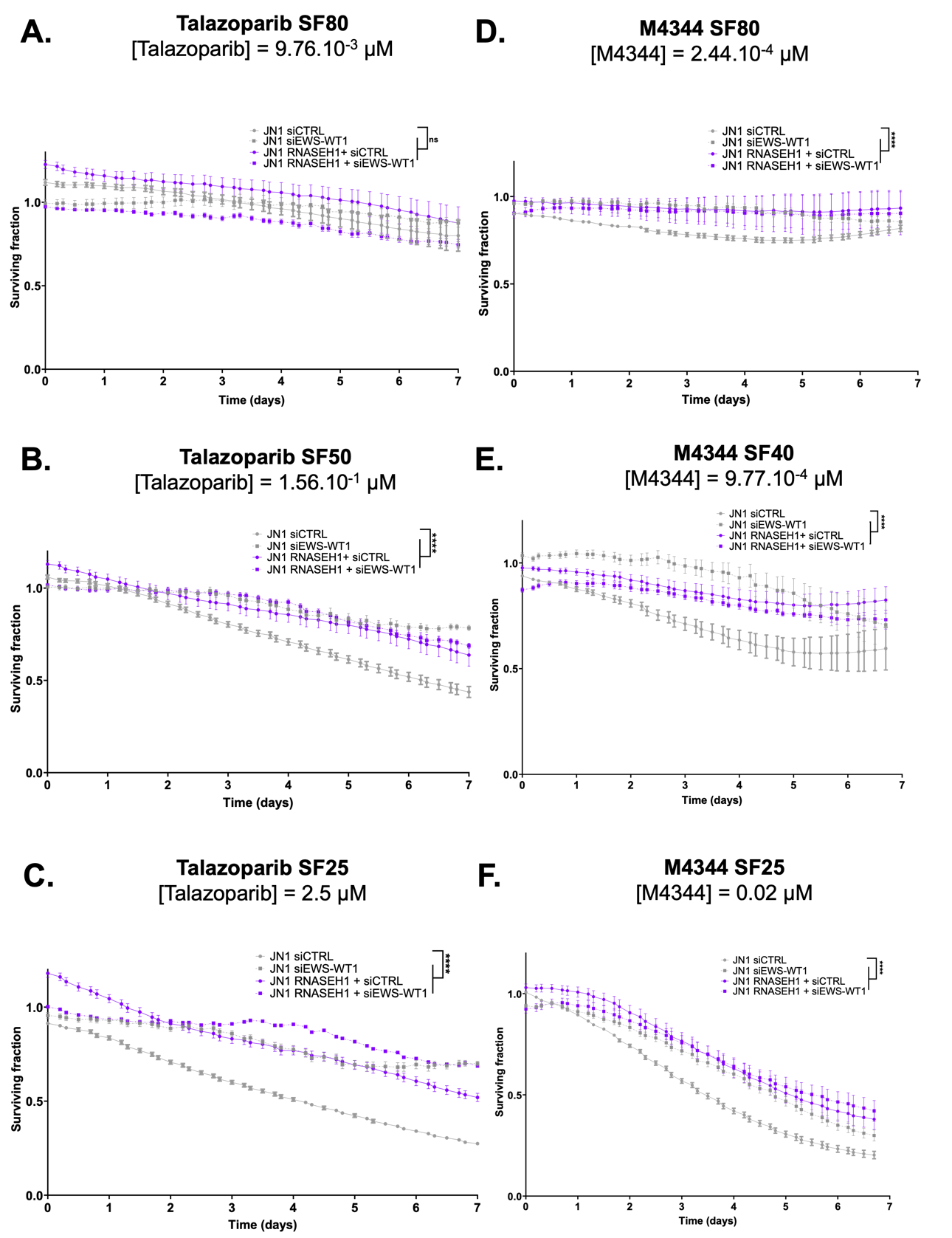
Supplementary Figure S17, related to Figure 5. Kinetic evaluation of the effect of RNase H1 overexpression on the sensitivity of JN1 cells to PARP and ATR inhibitors. A-C.** Incucyte®-generated survival curves of JN1 and JN1-RNaseH1 cells exposed to various talazoparib concentrations corresponding to the SF80 (A), SF50 (B) or SF25 (C) over 7 days, in presence or absence of siRNA-mediated silencing of EWS-WT1. Mean ± SD; *n* = 3, two-way ANOVA and *post hoc* Šídák’s test. **D-F.** Incucyte®-generated survival curves of JN1 and JN1-RNaseH1 cells exposed to various M4344 concentrations corresponding to the SF80 (A), SF40 (B) or SF25 (C) over 7 days, in presence or absence of siRNA-mediated silencing of EWS-WT1. Mean ± SD; *n* = 3, two-way ANOVA and *post hoc* Šídák’s test.

**Supplementary File. Full unedited membranes of the western blots and dot blots included in this study.**

**Full unedited membrane for Figure 3E**


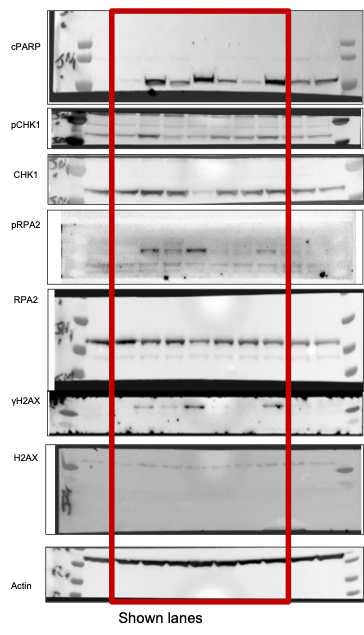


**Full unedited membrane for Figure 3F**


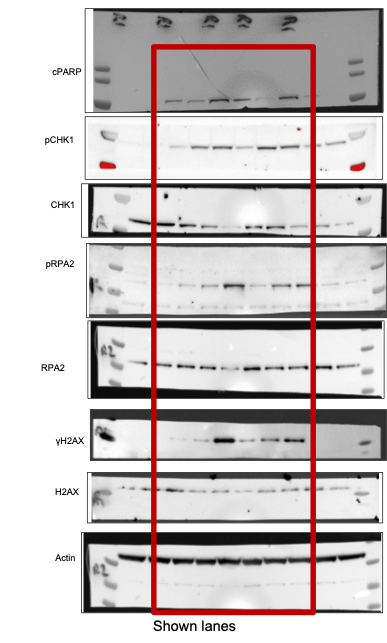


**Full unedited membrane for Figure 4A**


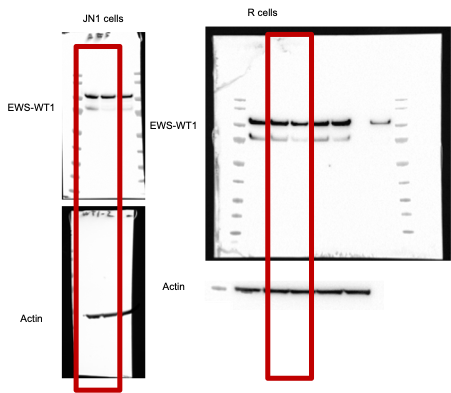


**Full unedited membrane for Figure 4H**


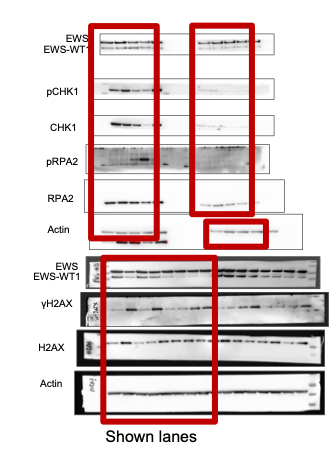


**Full unedited membrane for Figure 4I**


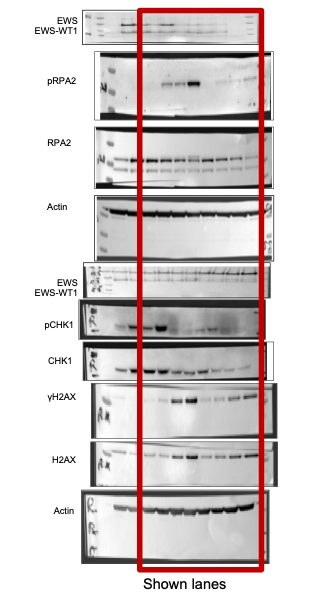


**Full unedited membrane for Figure 5C**


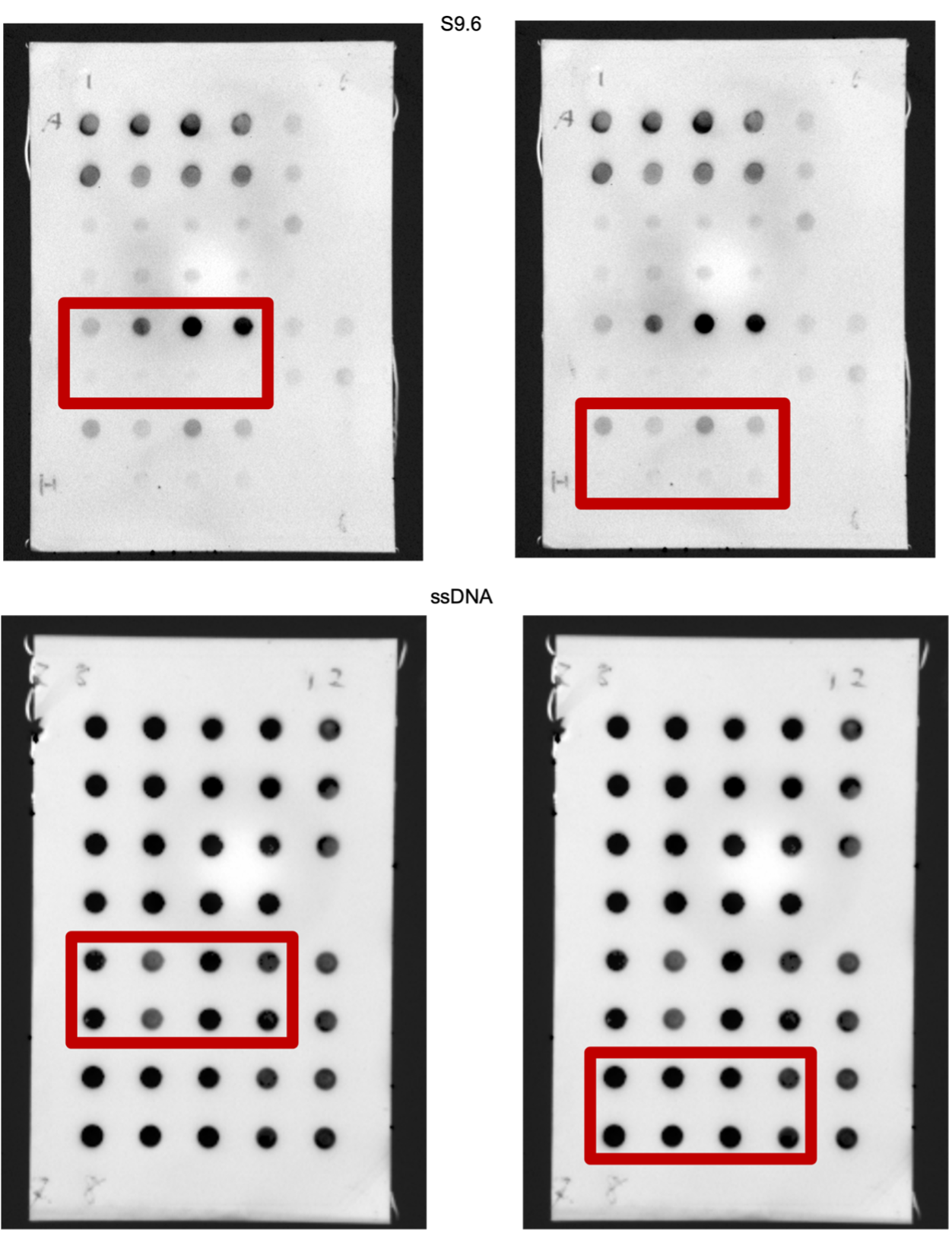


Shown Lanes. Shown Lanes

**Full unedited membrane for Figure 5D**


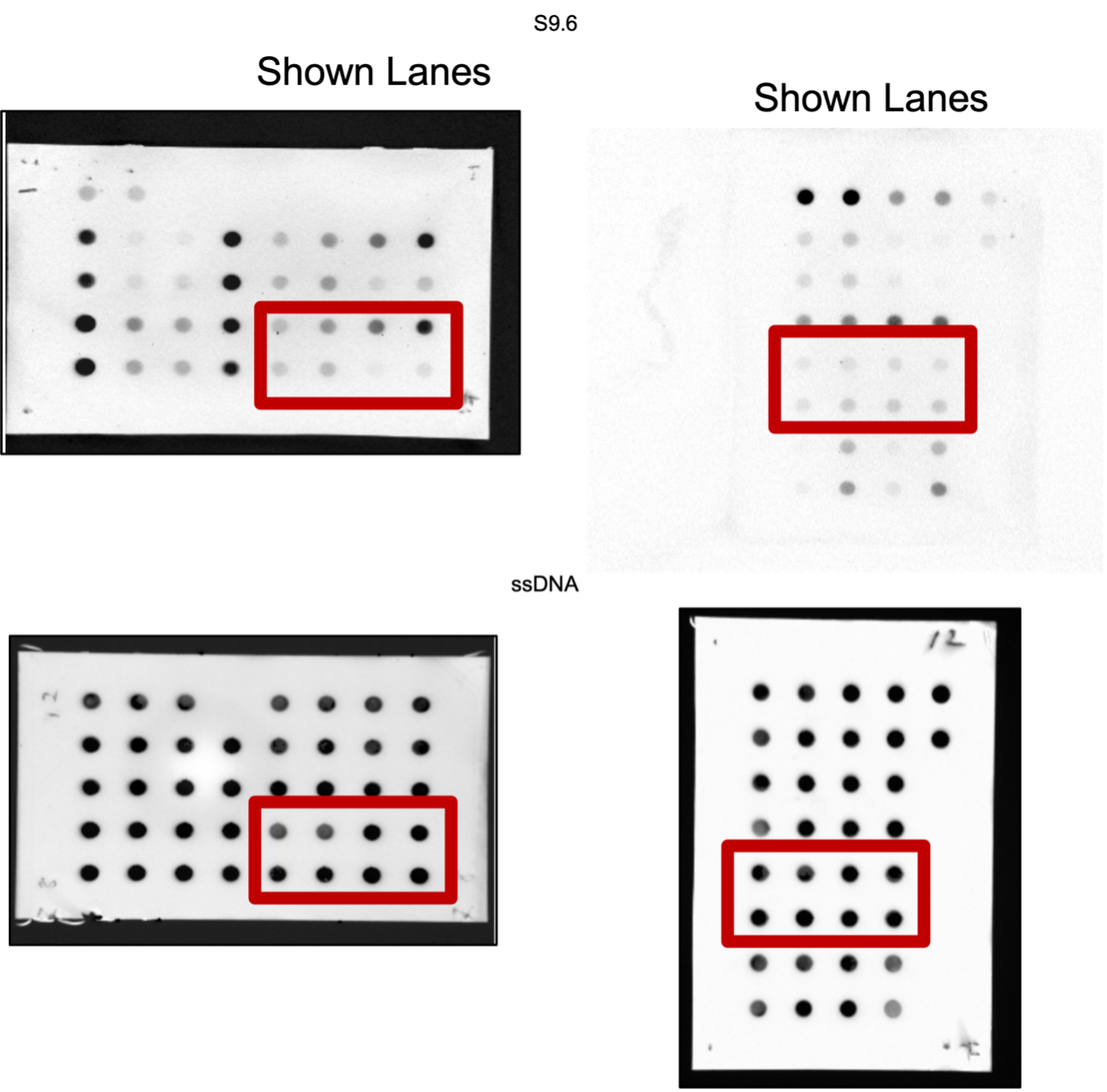


**Full unedited membrane for Figure 6A**


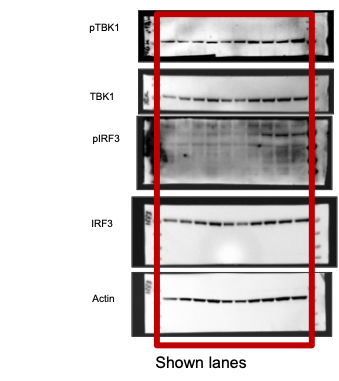


**Full unedited membrane for Figure 6E**
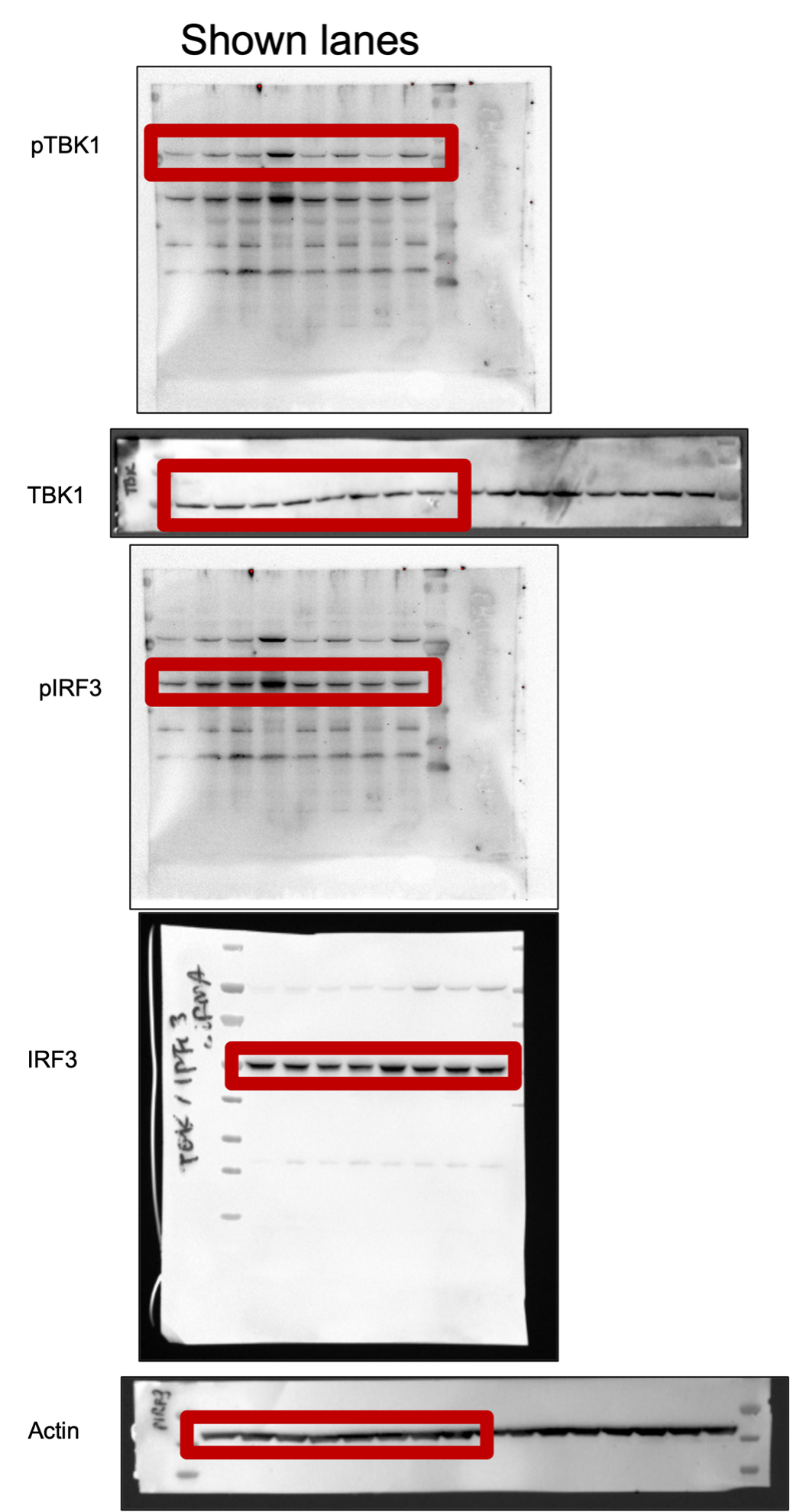


**Full unedited membrane for Figure S4A**


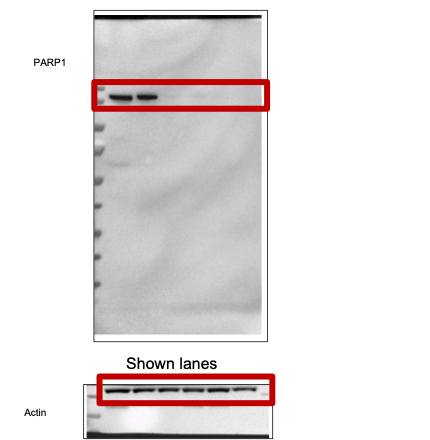


**Full unedited membrane for Figure S8C**


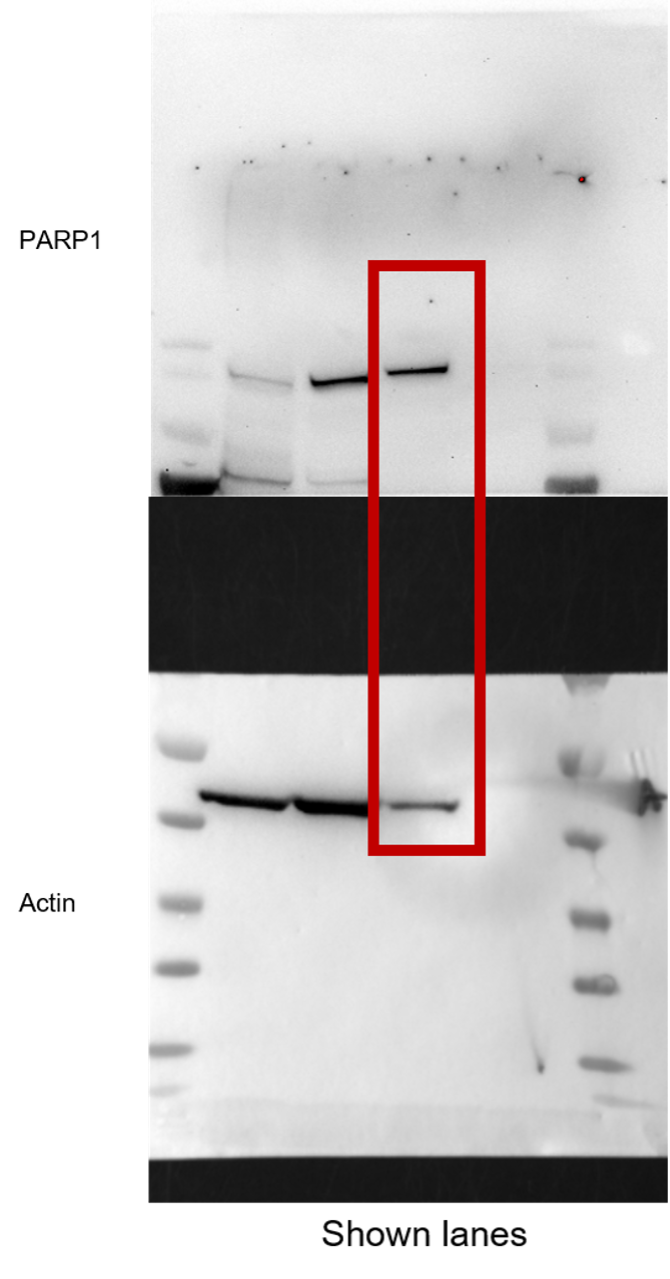


**Full unedited membrane for Figure S11E**


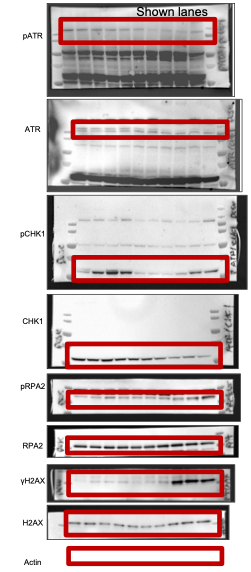


**Full unedited membrane for Figure S13G**


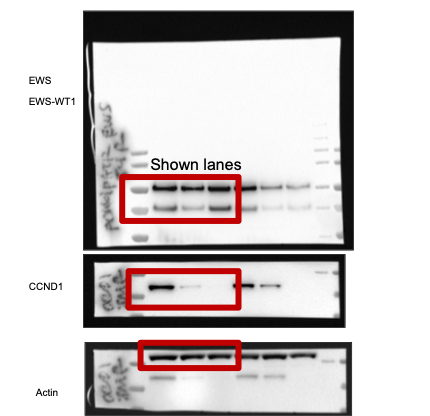


**Full unedited membrane for Figure S15B**


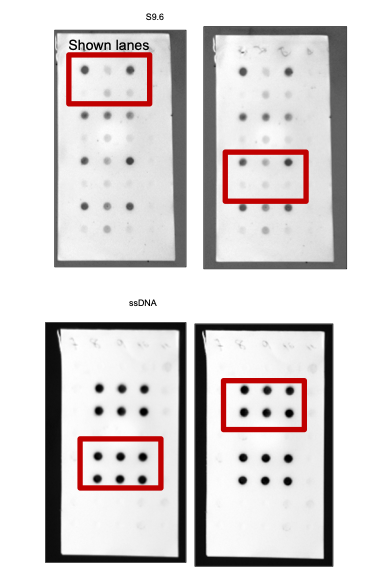


**Full unedited membrane for Figure S15C**


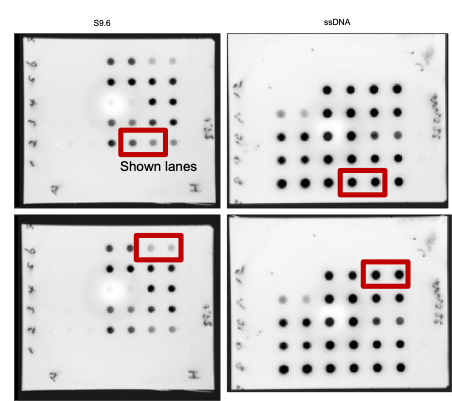

Supplement: Supplementary Data — Supplementary File containing methods, figures and uncropped membranes. [file can-23-3603_supplementary_data_suppsd.docx]
